# CAR-T cell manufacturing: Major process parameters and next-generation strategies

**DOI:** 10.1084/jem.20230903

**Published:** 2024-01-16

**Authors:** Melanie Ayala Ceja, Mobina Khericha, Caitlin M. Harris, Cristina Puig-Saus, Yvonne Y. Chen

**Affiliations:** 1 Department of Microbiology, Immunology, and Molecular Genetics, University of California−Los Angeles, Los Angeles, CA, USA; 2 Department of Medicine, University of California−Los Angeles, Los Angeles, CA, USA; 3 Jonsson Comprehensive Cancer Center, University of California−Los Angeles, Los Angeles, CA, USA; 4 Parker Institute for Cancer Immunotherapy Center at University of California−Los Angeles, Los Angeles, CA, USA; 5 Department of Chemical and Biomolecular Engineering, University of California−Los Angeles, Los Angeles, CA, USA

## Abstract

Chimeric antigen receptor (CAR)-T cell therapies have demonstrated strong curative potential and become a critical component in the array of B-cell malignancy treatments. Successful deployment of CAR-T cell therapies to treat hematologic and solid cancers, as well as other indications such as autoimmune diseases, is dependent on effective CAR-T cell manufacturing that impacts not only product safety and efficacy but also overall accessibility to patients in need. In this review, we discuss the major process parameters of autologous CAR-T cell manufacturing, as well as regulatory considerations and ongoing developments that will enable the next generation of CAR-T cell therapies.

## Teaser

The success of CAR-T cell therapies is dependent on effective cell manufacturing that impacts product safety, efficacy, and patient accessibility. Here, we discuss major process parameters of autologous CAR-T cell manufacturing, regulatory considerations, and emerging technologies in therapeutic cell manufacturing.

## Introduction

Since the first approval by the United States Food and Drug Administration (FDA) in 2017, chimeric antigen receptor (CAR)-T cell therapy has become a major component in the arsenal against B-cell malignancies including leukemia, lymphoma, and multiple myeloma (MM). To date, six products targeting either CD19 or B-cell maturation antigen (BCMA) have been approved in the U.S. (Cappell and Kochenderfer, 2023), with a large number of ongoing trials evaluating additional candidates targeting both hematological malignancies and solid tumors (Wang et al., 2023). In addition, CD19 CAR-T cell therapies have recently been applied to the treatment of autoimmune diseases, with early data showing promising outcomes for patients with systemic lupus erythematosus (Mackensen et al., 2022).

Successful expansion of CAR-T cell therapy to solid tumors and indications beyond cancer is highly dependent on the safety and efficacy of CAR-T cell products. CAR construct design and strategies by which CAR-T cells can be engineered to promote fitness, persistence, and antitumor efficacy have been reviewed in detail in several recent articles (Gao and Chen, 2022; Hamieh et al., 2023; Hou et al., 2021; Labanieh and Mackall, 2023). Here, we focus our attention on autologous CAR-T cell manufacturing, which plays a critical role in the clinical impact of CAR-T cell therapy by influencing the phenotype and function of the CAR-T cell products (Ceppi et al., 2022; Wang and Rivière, 2022). Although many of the aspects discussed in this review also apply to the manufacturing of allogeneic CAR-T cells, additional considerations exist for the engineering of donor T cells or the differentiation of stem cells into T-cell products, and discussions on these topics can be found in several excellent articles (Depil et al., 2020; Jing et al., 2022; Seet et al., 2017; Themeli et al., 2013; Ueda et al., 2023; van der Stegen et al., 2022).

The study of CAR-T cell manufacturing and its impact on therapeutic outcomes is inherently challenging due to the lack of perfectly controlled experiments. In the autologous cell therapy setting, each T-cell product is unique and donor-to-donor variation can be significant, especially in the context of heavily pretreated patients with prior exposure to hematologic toxicity and prior lymphotoxic therapies. Furthermore, manufacturing protocols vary from trial to trial and are often proprietary, precluding facile attribution of differences in clinical outcome to specific differences in manufacturing processes. Nevertheless, accumulating experience in the field has identified phenotypes that are correlated with durable responses to therapy, including a higher proportion of naïve and/or memory cell types (Bai et al., 2022; Chen et al., 2021), lower frequency of T cells expressing exhaustion markers (Finney et al., 2019), lower regulatory T cell (Treg) content (Good et al., 2022; Haradhvala et al., 2022), and higher overall proliferative potential (Fraietta et al., 2018a). Furthermore, clinical evidence suggests the CAR-T cell product’s phenotype also impacts the toxicity profile experienced by patients after infusion (Deng et al., 2020), highlighting the importance of manufacturing processes that can consistently yield cell products with the desirable characteristics.

**Figure 1.**
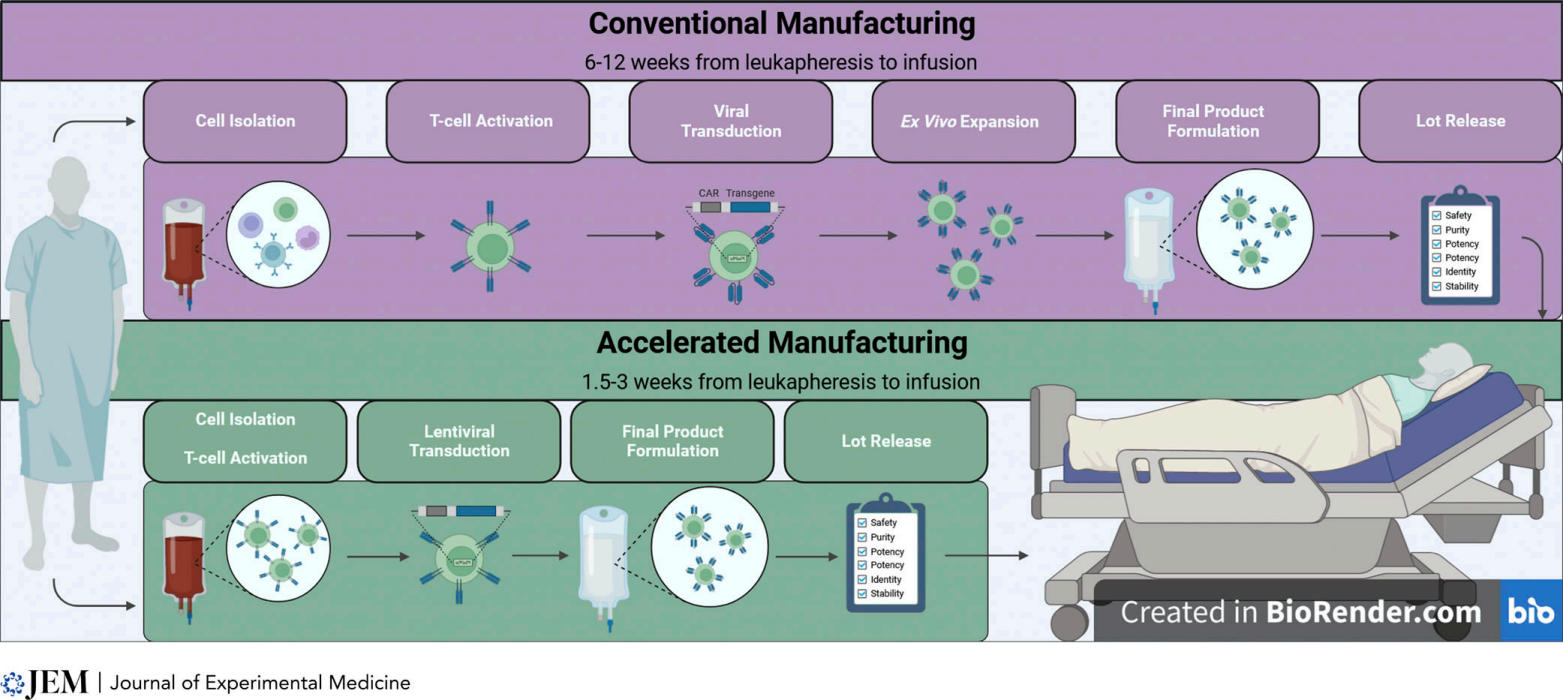
**Schematic of autologous CAR-T cell manufacturing processes.** Autologous CAR-T cell manufacturing generally involves initial cell isolation, T-cell activation, introduction of CAR transgene (or mRNA), cell expansion, and final formulation. Most products are cryopreserved and thawed at bedside prior to infusion into patients. Conventional manufacturing processes typically involve 1–2 wk of ex vivo cell manipulation and expansion, whereas abbreviated manufacturing processes can shorten the ex vivo period to 24–72 h. However, actual vein-to-vein time can be substantially longer due to the time required for transportation, product release testing, and clinical care considerations for the patient.

The manufacturing processes for several FDA-approved CAR-T cell products have been described in literature with broad similarities as well as unique features (Table 1). The overall process involves isolation of the starting cell population from the leukapheresis product, T-cell activation, genetic modification, ex vivo expansion, final product formulation, and product release testing (Fig. 1). This review aims to provide an overview of how process parameters in each key manufacturing step impact the resulting cell product and discusses next-generation manufacturing strategies with the potential to significantly alter the CAR-T cell therapy landscape in terms of therapeutic efficacy and patient access.

**Table 1.** Summary of FDA-approved CAR-T cell product manufacturing

Product name	Commercial name	Cell population prior to T-cell activation	Starting leukopak storage	Transgene integration method	Final product storage	References
Tisa-cel	Kymriah	Enriched T cells	Frozen	Lentivirus	Frozen	Fowler et al. (2022), Maude et al. (2018), Schuster et al. (2019), Tyagarajan et al. (2019)
Axi-cel	Yescarta	PBMCs (from Ficoll gradient enrichment)	Fresh	Retrovirus	Frozen	Jacobson et al. (2022), Locke et al. (2022), Roberts et al. (2018)
Brexu-cel	Tecartus	CD19-depleted and CD4/CD8-enriched T cells	Fresh	Retrovirus	Frozen	Mian and Hill (2021), Wang et al. (2020)
Liso-cel	Breyanzi	CD4 and CD8 T cells separately	Not reported	Lentivirus	Frozen	Kamdar et al. (2022), Sehgal et al. (2022), Teoh and Brown (2022)
Idecabtagene vicleucel	Abecma	PBMCs	Not reported	Lentivirus	Frozen	Al Hadidi et al. (2023), Hansen et al. (2023), Raje et al. (2019)
Ciltacabtagene autoleucel	Carvykti	Enriched T cells	Frozen	Lentivirus	Frozen	Berdeja et al. (2021); Committee for Medicinal Products for Human Use (2022); San-Miguel et al. (2023)

### Choosing the starting cell population

The choice of starting cell population for CAR-T cell manufacturing is an early decision point with significant impact on the production process as well as the final product. Autologous CAR-T cell manufacturing processes generally begin with mature T cells either in purified form or as part of the peripheral blood mononuclear cell (PBMC) population. Mature T cells differentiate from the naïve (T_N_) phenotype into stem-cell memory (T_SCM_), central memory (T_CM_), effector memory (T_EM_), effector (T_E_), and exhausted (T_EXH_) cells. Furthermore, T cells can be categorized into CD8^+^ cytotoxic T cells, CD4^+^ helper T cells, and CD4^+^ Tregs. Within the helper T cell category, one could further identify subpopulations such as Th1, Th2, Th17, and follicular helper cells. The diversity of T-cell phenotypes renders the choice of starting cell population a major process parameter in CAR-T cell manufacturing (Golubovskaya and Wu, 2016). However, biology is not the only factor influencing the choice of starting material as the patient’s clinical history and health status also impose practical constraints on the materials that can be feasibly obtained for cell manufacturing.

#### CD4^+^ versus CD8^+^ T cells

CAR-T cells are activated directly through CAR antigen binding without the need for coreceptor binding to MHC molecules. Therefore, CAR-T cell function does not, in principle, require CD4 and CD8 molecules as coreceptors. Early developmental work on CAR-T cells contemplated the advantage of selective enrichment of CD8^+^ T cells to maximize cytotoxicity (Berger et al., 2008). However, CD4^+^ helper T cells also play a critical role in promoting CD8^+^ T cells’ effector function, expansion, and persistence (Kumamoto et al., 2011), and it has been shown that the presence of CD4^+^ T cells in the tumor microenvironment results in increased recruitment, proliferation, and function of CD8^+^ cytotoxic T cells (Bos and Sherman, 2010). Therefore, currently approved CAR-T cell products all contain both CD4^+^ and CD8^+^ T cells, although divergent manufacturing approaches are employed.

Among the CD19-specific CAR-T cell products, tisagenlecleucel (tisa-cel), axicabtagene ciloleucel (axi-cel), and brexucabtagene autoleucel (brexu-cel) all utilize mixed CD4^+^ and CD8^+^ T cells (Mian and Hill, 2021; Roberts et al., 2018; Tyagarajan et al., 2019). In contrast, lisocabtagene maraleucel (liso-cel) comprises two different cell products—made separately from CD4^+^ and CD8^+^ starting cell populations—that are admixed prior to administration (Teoh and Brown, 2022). This more complex manufacturing process is motivated by preclinical studies suggesting defined CD4:CD8 ratios may confer superior antitumor efficacy (Sommermeyer et al., 2016), although clear distinctions in efficacy and safety profiles between liso-cel and other products will require broader and longer-term data collection (Abramson et al., 2020, 2023; Locke et al., 2022; Schuster et al., 2021).

Cell isolation is commonly achieved through magnetic bead–based cell sorting. A mixture of CD4^+^ and CD8^+^ cells can be isolated through the depletion of non-T cells or by positive selection using a combination of CD4^−^ and CD8-binding microbeads. The process of isolating a mixed T-cell population is relatively streamlined and can be accomplished at lower cost compared with parallel manufacturing of separate CD4^+^ and CD8^+^ cell cultures, but it precludes the possibility of precisely controlling the CD4:CD8 ratio in the final product. Alternatively, CD4^+^ and CD8^+^ T cells can be isolated and cultured separately, allowing for the formulation of a final product with precise CD4:CD8 ratios. However, this approach comes at the cost of increased labor, time, and cost of manufacturing. Furthermore, optimal CD8^+^ T-cell expansion requires the presence of CD4^+^ T cells (Castellino and Germain, 2006), rendering the manufacturing of isolated CD8^+^ T cells vulnerable to production failure. A potential solution to this challenge is to isolate CD4^+^ and CD8^+^ T cells separately but coculture at defined starting ratios, with the aim of generating a final cell product with approximately the desired ratio of CD4:CD8 T cells. Such a strategy has been shown to overcome the difficulty of CD8^+^ T-cell expansion, resulting in a product with increased cytotoxic function in mice compared to CD4^+^ and CD8^+^ T cells cultured separately (Lee et al., 2018). However, such “defined” manufacturing methods cannot completely prevent manufacturing failures. Importantly, the starting CD4:CD8 ratio needed to yield the desired final ratio in the product can vary by donor, thus a precise product profile remains challenging by this approach.

#### T-cell differentiation status

Beyond CD4:CD8 ratios, the differentiation state of the T-cell product has been shown to correlate with antitumor efficacy profiles (Bai et al., 2022; Chen et al., 2021; Fraietta et al., 2018a; Haradhvala et al., 2022). Upon antigen stimulation, T cells undergo clonal expansion and a subset differentiates into T_E_ cells, which have potent cytotoxicity and cytokine production capabilities but are relatively short-lived (Golubovskaya and Wu, 2016), in contrast, a subset of activated T cells become memory T cells that are long-lived and quickly activated in response to a secondary antigen challenge (Sallusto et al., 2004). Compared with terminally differentiated T_E_ cells, T_N_, T_SCM_, and T_CM_ cells possess greater potential for long-term persistence and produce lower levels of inflammatory cytokines, which could together enable durable anti-tumor response while avoiding severe acute toxicities such as cytokine release syndrome (CRS; Chang et al., 2014; Farber et al., 2014; Kishton et al., 2017). Such findings have supported exploration of manufacturing processes that begin by enriching for naïve and memory T cells, with early evidence suggesting such products can achieve robust efficacy combined with a favorable safety profile (Larson et al., 2023; Wang et al., 2016).

The surface expression of CD45RA, CD45RO, CD62L, and CCR7 is commonly used to identify T-cell subtypes (Golubovskaya and Wu, 2016), and naïve and memory T cells can be isolated from a leukapheresis product by selecting for CD62L^+^ cells (Arcangeli et al., 2022; Casati et al., 2013; Larson et al., 2023). However, CD62L^+^ selection can simultaneously lead to unintended enrichment of CD14^+^ myeloid cells and CD25^+^ Tregs that also express CD62L (Larson et al., 2023). Myeloid cells have been shown to significantly reduce the efficiency of T-cell activation and subsequent viral transduction, potentially due to phagocytosis of the anti-CD3/CD28 magnetic beads used to activate T cells (Künkele et al., 2019; Stroncek et al., 2016; Wang et al., 2021a). Furthermore, the presence of Tregs in the CAR-T product has been reported to correlate with poor antitumor efficacy (Beider et al., 2019; Colombo and Piconese, 2007; Haradhvala et al., 2022). These findings suggest that CAR-T products may benefit from the depletion of CD14^+^ and CD25^+^ cell types prior to the enrichment of CD62L^+^ cells, and the use of CD14^−^/CD25^−^/CD62L^+^ cells as the starting material for CAR-T cell manufacturing was evaluated in multiple clinical trials (e.g., NCT02208362 and NCT04007029).

Unexpectedly, data from one trial (NCT04007029) revealed that CAR-T cell products made from starting populations that were not depleted of CD14^+^ and CD25^+^ cells prior to CD62L enrichment had similar levels of activation, transduction, expansion, and final CD3^+^ purity level compared with products made from CD14/CD25-depleted starting populations (Larson et al., 2023). Products made from non-CD14/CD25–depleted cells showed a clear enrichment in CD4^+^ T cells at the end of cell manufacturing, but no significant difference in therapeutic outcome was noted between cell products made from CD14/CD25-depleted or non-depleted starting cell populations based on the small number of patients in this phase 1 trial. Of note, the cell-manufacturing process used in this particular clinical trial utilized a polymeric nanomatrix T-cell stimulant rather than a magnetic bead–based activating reagent, and the differential phagocytic response displayed by myeloid cells against activating reagents of different sizes and rigidities may have played a key role in the behavior of the resulting cell product. We next discuss the importance of the activation step in the CAR-T cell manufacturing process.

### T-cell activation methods

T-cell activation is critical to the success of the cell-manufacturing process as it directly impacts the efficiency of CAR transgene integration (discussed in the next section) as well as T-cell expansion during ex vivo culture. T-cell activation is most commonly achieved through the stimulation of CD3 and CD28 combined with cytokine support. CD3 signaling (signal 1) triggers T-cell activation while CD28 signaling provides the necessary costimulation (signal 2) to avoid anergy (Mescher et al., 2006). In addition, cytokine cocktails (signal 3)—most commonly IL-2, IL-7, and/or IL-15—are used to support T-cell expansion (Arcangeli et al., 2020; Künkele et al., 2019). At present, manufacturing protocols typically utilize magnetic beads or colloidal polymeric nanomatrices coated with anti-CD3 and anti-CD28 antibodies to provide signals 1 and 2. Magnetic beads offer solid support that mimics target cells presenting peptide–MHC complexes and costimulatory ligands for T-cell engagement, and anti-CD3/CD28 beads can simultaneously serve as a T-cell isolating agent for magnetism-based cell sorting. However, magnetic beads are prone to engulfment by myeloid cells (Wang et al., 2021a), thus cell sorting using anti-CD3/CD28 beads poses the risk of enriching for CD14^+^ cells while reducing the amount of activating reagents available to stimulate T cells. Furthermore, a debeading step through magnetic separation is required to generate a pure CAR-T cell product prior to reinfusion. In contrast, colloidal polymeric nanomatrices appear to be less prone to elimination by myeloid cells (Larson et al., 2023) and can be removed by simple centrifugation. However, head-to-head comparisons of these reagents have not been reported to enable rigorous comparison of resulting cell products.

Regardless of format, commercially available activating agents are standardized products applied at a fixed bead:cell ratio or per-volume dilution ratio. More recent developments have explored the possibility of personalized T-cell activation reagents tailored for each individual product based on the patient’s disease type. For example, utilizing mesoporous silica microrods coated with a lipid bilayer, Mooney and colleagues developed APC-mimetic scaffolds that can not only provide anti-CD3 and -CD28 signals but also enable sustained release of IL-2 (Cheung et al., 2018). The density of anti-CD3 and -CD28 antibodies can be precisely tuned to provide the optimal stimulation intensity to maximize T-cell fitness (Zhang et al., 2023). The implementation of finely tuned, highly personalized reagents would have to be balanced against practical considerations of production throughput and process robustness. Nevertheless, the emergence of “smart” materials that can be adapted to different product requirements could significantly expand the flexibility and quality of manufacturing processes.

It should be noted that although T-cell activation is an indispensable step in present-day CAR-T cell manufacturing, next-generation manufacturing processes are contemplating the possibility of eliminating T-cell activation. For example, a recent report described the successful transduction of non-activated T cells by lentiviral vectors (Ghassemi et al., 2022). Next, we discuss the various methods available for CAR transgene introduction, including both viral and non-viral approaches.

### Introducing the CAR transgene

The method of CAR transgene delivery can significantly impact the level of CAR transgene expression as well as genotoxicity, which in turn influences the safety and efficacy of the resulting CAR-T cell product. In this section, we broadly evaluate both viral and non-viral gene-delivery methods for CAR-T cell manufacturing.

#### Viral gene delivery

Currently, all FDA-approved CAR-T cell products use lentiviral or retroviral transduction to achieve CAR transgene integration (Labbé et al., 2021; Table 1). Viral transduction benefits from relatively high integration efficiency, and T cells expressing stably integrated CAR constructs have been shown to persist for >10 yr after T-cell infusion (Melenhorst et al., 2022; Scholler et al., 2012). However, virally integrated transgenes lack insertion-site specificity, presenting a theoretical risk of insertional mutagenesis. A genome-wide profiling study compared transgene integration sites in CAR-T cell products made with γ-retroviral vectors versus lentiviral vectors, with results indicating that lentiviral vectors are more likely to integrate in the intron and intergenic regions compared with retroviruses; in contrast, retroviruses have higher frequencies of integration into promoters, untranslated regions, and exon regions, resulting in a greater impact on mRNA transcript levels (Shao et al., 2022). It is important to note that there has been no reported instance to date of viral integration resulting in oncogenic transformation of CAR-T cells (Cornetta et al., 2018; Lyon et al., 2018; Marcucci et al., 2018). On the contrary, a case study reported a patient whose response to CD19 CAR-T cell therapy appeared to correlate with the expansion of a single CAR-T cell clone whose CAR transgene was randomly inserted in the *TET2* locus, thereby knocking out the only functional *TET2* copy as the patient had a congenital missense mutation in the other *TET2* allele (Fraietta et al., 2018b). Similarly, clonal expansion of CD22 CAR-T cells with a copy of the provirus integrated in the *CBL* gene locus was found to expand dramatically prior to eradication of residual disease in a patient with B-cell acute lymphoblastic leukemia (B-ALL; Shah et al., 2019a). Although these cases show that random insertion may serendipitously result in positive therapeutic outcome, the risk of insertion into undesirable loci remains a motivation for exploring alternative transgene-integration strategies that could ensure site specificity.

Aside from integration-site considerations, viral vectors present another potential risk in the form of replication-competent retroviruses (RCRs) and lentiviruses (RCLs). Accordingly, the FDA requires RCR/RCL detection assays for the viral vectors as well as virally transduced cell products (Center for Biologics Evaluation and Research, 2020). However, there has been no reported instance of RCR and RCL detected in clinical CAR-T cell products to date, and balancing the cost and potential delays caused by RCR/RCL testing with the practical benefit of such assays remains a topic of active scientific exploration (Cornetta et al., 2018; Lyon et al., 2018; Marcucci et al., 2018).

Although viral transduction can in principle achieve high transduction efficiency, achieving consistent levels of transgene integration in patient cell products presents a technical challenge. Indeed, 10% or lower CAR positivity—levels set based on the premise that transduced cells will expand in vivo upon antigen exposure—is not uncommon as a minimum threshold for transduction efficiency in cell products used in clinical trials (O’Rourke et al., 2017; Stadtmauer et al., 2020; Tong et al., 2020). To minimize the risk of manufacturing failure due to poor transgene expression, manufacturing protocols can incorporate the use of transduction enhancers such as protamine sulfate, retronectin, and poloxamer (Cornetta and Anderson, 1989; Delville et al., 2018; Rajabzadeh et al., 2021). A rate-limiting step in viral transduction is the initial attachment of the viral particle to the cell membrane, a process that can be facilitated by the use of retronectin for retroviral transduction and polycations such as protamine sulfate for lentiviral transduction. Polycations serve as an electrostatic bridge that links the virus to the cell surface (Doms, 2016). However, protamine sulfate’s toxicity to T cells presents a counterweight to the benefit of increased transduction efficiency. In contrast, retronectin does not lead to T cell toxicity but its use is limited to retroviral vectors. Proprietary transduction enhancers with more favorable toxicity profiles have been developed (Delville et al., 2018), although their high costs and proprietary access can present a barrier to widespread usage.

In addition to the biological properties of virus transduction, practical considerations also influence the use of viruses in CAR-T cell manufacturing. The number of certified facilities capable of producing clinical-grade virus remains limited. Consequently, the time and financial cost required for virus production can present a bottleneck in both investigational drug development and commercial CAR-T cell manufacturing, highlighting the appeal of alternative, non-viral delivery systems that may be more flexible and cost-effective (Balke-Want et al., 2023).

#### Non-viral gene delivery

Several different non-viral gene delivery methods have been explored in the context of CAR-T cell manufacturing, including CRISPR/Cas9, transposons, and mRNA transfection. CRISPR/Cas9 has enjoyed widespread adoption as an efficient method for genome modification, and its potential to enhance CAR-T cell therapies has not gone unnoticed (Dimitri et al., 2022). To achieve site-specific gene modification, the Cas9 nuclease is complexed with a single-guide RNA (sgRNA), identifies the target site on genomic DNA via sequence complementarity to the sgRNA, and introduces a double-stranded DNA break. Next, transgene insertion is facilitated through homologous recombination between a DNA template that encodes for the desired transgene (e.g., CAR) and the genomic cut site (Jiang and Doudna, 2017).

The ability to specify the transgene-integration site opens up new possibilities in engineering T cells with desired functions. A number of extragenic “safe-harbor” sites have been computationally identified and empirically tested to support the precise genetic modification of T cells for therapeutic functions (Odak et al., 2023). In particular, it has been shown that CAR transgenes can be efficiently integrated into the *TRAC* locus that encodes for the endogenous TCR α chain using an adeno-associated viral vector as the homology-directed repair template (Eyquem et al., 2017). This strategy commandeers the endogenous gene regulatory mechanisms that dynamically control TCR expression levels in response to T-cell activation states, with data suggesting that the resulting CAR-T cells may be more efficacious than virally integrated CAR-T cells (Eyquem et al., 2017). In addition, non-viral CRISPR/Cas9-based gene-editing strategies have also been used to replace the endogenous TCR with tumor-specific TCRs, enabling the development of highly personalized T-cell therapies (Foy et al., 2023; Puig-Saus et al., 2023). As another example, the aforementioned case study involving *TET2* mutations has inspired subsequent evaluation of intentional CAR transgene integration into the *TET2* site as a means to enhance CAR-T cell proliferation and persistence (Jain et al., 2023). Interestingly, results showed that the benefit of *TET2* disruption on in vivo antitumor efficacy varied with the specific CAR constructs expressed, echoing similar findings on CAR integration into the *TRAC* locus (Zah et al., 2020) and highlighting the non-trivial nature of identifying optimal integration sites for CAR transgenes.

In addition to facilitating site-specific transgene integration, CRISPR/Cas9 has also been used in combination with viral integration to knock out undesirable endogenous genes while non-site-specifically integrating the transgene encoding for a tumor-targeting receptor. For example, the first FDA-approved clinical trial involving CRISPR/Cas9-edited T cells utilized a manufacturing process that knocked out PD-1 and the endogenous TCR with CRISPR/Cas9 while lentivirally integrating a transgenic TCR targeting NY-ESO-1 (Stadtmauer et al., 2020). Of note, it was observed that the frequency of NY-ESO TCR-expressing cells with mutations in the *PDCD1* locus decreased over time after infusion, suggesting that PD-1–edited T cells might lack the ability to form long-term memory (Stadtmauer et al., 2020). In another phase 1 clinical study, CRISPR/Cas9 was used to knock out PD-1 and the TCR α chain while a mesothelin-targeting CAR was lentivirally integrated (Wang et al., 2021b). Although both studies showed acceptable safety profiles, neither resulted in dramatic improvements in efficacy, highlighting the need for continued improvement. Furthermore, CRISPR/Cas9-mediated double-stranded DNA break poses a non-trivial risk of unintended genomic changes that require careful analysis and quality control, and a recent study revealed the potential for chromosome loss in Cas9-engineered CAR-T cells (Tsuchida et al., 2023). Interestingly, the same study observed that the specific order of operations impacted the frequency of chromosome loss, which can be reduced if the Cas9-mediated double-stranded break is performed before the T cells are activated.

As an alternative to CRISPR/Cas9, transposons have also been used to achieve stable CAR transgene integration into T-cell products. In particular, piggyBac and Sleeping Beauty (SB) transposon systems have been evaluated in the clinical setting (Monjezi et al., 2017; Prommersberger et al., 2021; Zhang et al., 2021). Transposon systems perform “cut-and-paste” processes in which a transposase binds to terminal inverted repeat sequences flanking a target gene element (i.e., the transposon), excises the transposon through double-stranded DNA break, and reintegrates the transposon into a suitable genomic site (e.g., palindromic sequences comprising AT dinucleotides in the case of SB transposons; Sandoval-Villegas et al., 2021; Vigdal et al., 2002). Transgene integration by this means is not site specific, and piggyBac transposases have been reported to exhibit preferential insertion into transcriptional start sites similar to retroviruses (Gogol-Döring et al., 2016), again raising the potential risk of insertional mutagenesis. In a phase 1 clinical trial, two patients treated with allogeneic CD19 CAR-T cell products generated with the piggyBac transposon system developed CAR-T cell–derived lymphoma, resulting in one fatality (Bishop et al., 2021). Post-hoc analysis indicated that the malignant CAR-T cells did not contain transgene insertion into known oncogenic sites but displayed significant copy-number gains and losses of multiple chromosomes as well as transcriptional readthrough from the transgene promoter (Micklethwaite et al., 2021). Importantly, the products that resulted in malignant transformation had unusually high vector copy numbers (VCN), with one product having a VCN of 25 (Schambach et al., 2021). By comparison, in the absence of product-specific justifications, the FDA-recommended maximum is five copies per transduced cell for virally transduced cell products (Center for Biologics Evaluation and Research, 2022b).

One strategy to eliminate the risk of oncogenic insertion is to avoid stable integration of the CAR-encoding transgene and instead transiently express the CAR from an mRNA template (Yoon et al., 2009). In addition to eliminating the risk of genotoxicity, transient CAR expression from mRNA has also been explored as a means to reduce potential toxicity, particularly when the CAR targets an antigen that is also present in healthy tissues (Zhao et al., 2010). Transgene-encoding mRNA templates are typically delivered into T cells by electroporation, and transgene expression lasts for ∼1 wk, with expression levels declining each day after electroporation (Yoon et al., 2009; Zhao et al., 2006). Therefore, the potential safety advantage of transient gene expression must be balanced against the necessarily short-lived nature of the therapy. Clinical evaluations suggest that mRNA-encoded CAR-T cells are safe and can exhibit antitumor efficacy (Beatty et al., 2014; Zhao et al., 2010), but the ability to achieve complete and durable tumor control remains an area of investigation.

### Ex vivo cell expansion

Once T cells have been modified to express the CAR transgene, the product must be expanded to a sufficiently large number to meet the required dose for administration. The duration of this expansion period varies across different protocols, but typical processes last 1–2 wk from the time of T-cell activation to the time of cell harvest. The expansion condition is optimized for T-cell growth, with cytokine support such as IL-2, IL-7, and/or IL-15 (Arcangeli et al., 2020; Künkele et al., 2019). Although the main purpose of the expansion period is to increase T-cell numbers, it also eliminates non-T cells by exposing them to culture conditions that are suboptimal to other cell types, thus enabling the generation of a highly enriched T-cell product even if the manufacturing process begins with mixed PBMCs. In addition to cytokines, culture supplements such as FBS also play a critical role in supporting T-cell expansion, although defined media compositions that enable more precise formulation and lower risk of zoonotic pathogens are rapidly supplanting components such as FBS (Center for Biologics Evaluation and Research, 2022a). Finally, recent studies have explored the use of various pharmacological supplements to promote desirable T-cell phenotypes and enhance the therapeutic potential of the resulting CAR-T cell product. For example, the tyrosine kinase inhibitor dasatinib has been used in CAR-T cell manufacturing to inhibit CAR tonic signaling (i.e., receptor signaling in the absence of antigen stimulation), thereby preventing premature T-cell exhaustion and increasing CAR-T cell functionality (Weber et al., 2021). This strategy was used to produce GD2-targeted CAR-T cells—which are known to strongly tonically signal and exhibit a propensity toward exhaustion (Long et al., 2015)—for a phase 1 trial, with early results showing remarkable improvements in multiple patients with H3K27M-mutated diffuse midline gliomas (Majzner et al., 2022). Since multiple doses of CAR-T cells were administered to each patient enrolled in this trial, definitive conclusions on whether dasatinib prevented exhaustion and/or enabled sustained T-cell function remain elusive. Nevertheless, this example demonstrates the feasibility of pharmacologically modulating the CAR-T cell manufacturing process to generate functional CAR-T cell products for clinical translation.

### Product release testing

Capitalizing upon the groundbreaking success of CAR-T cells in the treatment of hematological malignancies in 2017, over 1,000 active CAR-T cell clinical trials are in progress globally, concentrated in the North American and Eurasian continents (Wang et al., 2023). This boom in clinical investigations has refined our understanding of relevant parameters for successful CAR-T cell products. As with any proposed therapy, CAR-T cells seeking clinical evaluation must meet defined product characteristics relating to safety, purity, potency, identity, and stability. All FDA-approved CAR-T cell products (Havert, 2017; Liu, 2017; Price, 2020; Kwilas, 2021; Schultz, 2021; Ye, 2022) measure the bolded product characteristics below using combinations of the following metrics:

•**Safety:** Mycoplasma, sterility, endotoxin, residual viral-agent quantification, viability, and CAR transgene quantification
•**Purity:** T-cell purity, viability, residual reagent quantification, transduction efficiency, and presence of contaminating tumor cells
•**Potency:** Transduction efficacy, viability, CAR expression, cytotoxicity, or cytokine (e.g., IFN-γ) production upon antigen stimulation
•**Identity:** CAR expression, visual appearance, clarity, dose, and viability
•**Stability:** Formulation, shipping, and storage.

The FDA has recently drafted standardized expectations for CAR-T cell products. In particular, lot release criteria for early-phase investigational new drug application submissions do not require validated potency assays and specifications, but such assays must be included when generating data in support of the Biologics License Application (Center for Biologics Evaluation and Research, 2022a; Dias et al., 2023).

Based on accumulating data from ongoing clinical trials and real-world experience, additional parameters have also been proposed to propel future development of more reproducible, effective, and safe therapeutics. For instance, given evidence suggesting CAR-T products with less differentiated, more memory-like phenotypes have greater potential to achieve durable tumor clearance (Chen et al., 2021), future commercial products may benefit from setting an efficacy characteristic metric that specifies the proportion of favorable T-cell subpopulations within the product. However, altering and tightening product specifications must be guided by rigorous scientific evidence and fundamental biological understandings to avoid triggering unnecessary manufacturing failures that could curtail access to therapy for patients.

In certain instances of product failure involving non-life-threatening lot release criteria (e.g., dose, transduction efficacy, cytokine production level, or CAR expression), out-of-specification products can be administered to patients upon receiving necessary regulatory approvals. Within expanded-access protocols, patients have received out-of-specification commercial and clinical-trial products and experienced comparable clinical outcomes as patients treated with standard products (Chong et al., 2019; Jacobson et al., 2020; Rossoff et al., 2020; Schultz et al., 2022). Accumulating clinical experience with CAR-T cell products in the real-world setting will be invaluable in understanding which product characteristics truly impact patient safety and therapeutic efficacy, and may serve as a guide for further refinement of regulatory guidance on CAR-T cell product release testing.

### Next-generation strategies for CAR-T cell manufacturing

The manufacturing strategies discussed thus far have supported the development of multiple CAR-T cell products to date and enabled the demonstration of CAR-T cell therapies’ potential to overcome advanced malignancies. However, real-world experiences with CAR-T cell therapy after FDA approval have also highlighted the limitations of conventional cell-manufacturing processes, which are low throughput and resource intensive, resulting in limited patient access to potentially life-saving therapies (Levine et al., 2016). As of early 2023, 90% of patients with MM experience disease progression and 25% of patients succumb to disease while waitlisted for BCMA-directed CAR-T cell product slots, with waiting periods ranging from 1 to 10 mo before undergoing apheresis (Al Hadidi et al., 2023; Kourelis et al., 2023). Upon apheresis, the cells must undergo transportation to and from the manufacturing site, ex vivo modification and expansion, and stringent quality-control testing (Al Hadidi et al., 2023; Hansen et al., 2023; San-Miguel et al., 2023). The majority of patients required bridging therapy to combat further clinical deterioration during cell manufacturing (Al Hadidi et al., 2023; San-Miguel et al., 2023), which could lead to further infusion delays in patients who experience adverse reactions related to bridging (Roddie et al., 2023; Shahid et al., 2022).

Even in the absence of medical complications during the vein-to-vein time, 4–7% of patients are unable to receive their CAR-T cell products as a result of manufacturing failures (Bhaskar et al., 2021; St Martin et al., 2023), with risk factors including reduced fitness of patient T cells following multiple lines of treatment and the lengthy manufacturing process itself (Jo et al., 2023). Therapeutic options following unsuccessful product manufacturing include repeating apheresis or transitioning to alternative therapies, though survival outcomes are poor in both cases (Jagannath et al., 2021; Jo et al., 2023; Mateos et al., 2023). Altogether, the multiple sources of delays leading to loss of life underscore the criticality of timely access to CAR-T cell products (Chen et al., 2022). Here, we discuss next-generation strategies under development to address key challenges in CAR-T cell manufacturing (Table 2).

**Table 2.** Next-generation CAR-T cell manufacturing strategies

	Pros	Cons
Accelerated cell manufacturing	•Reduced vein-to-vein time •Less resource intensive •Increased manufacturing capacity •Less differentiated T cells in final product •Potential for reduced T-cell doses	•Increased potential for contaminating tumor cells in product (particularly for hematological malignancies) •Potential for T cells having overly active phenotype, leading to increased toxicities •Complexity in product-release testing (e.g., inability to distinguish between transient protein expression from pseudo-transduction versus stable integration)
Process automation	•Reduced personnel and infrastructure costs •Reduced probability of human error •Potential for on-site manufacturing enabling fresh cell products	•Reduced capability to respond to patient-specific cell behaviors during manufacturing •Challenge in ensuring consistency across multiple sites for point-of-care manufacturing •Limited capacity to perform long-term release testing for fresh products
In vivo cell manufacturing	•Cost and time saving from eliminating need for ex vivo cell manufacturing •Potential for less differentiated T cells •Off-the shelf reagents instead of patient-specific products	•Potential genotoxicity and immunogenicity •Potential transgene insertion into non-T cells •Unknown safety profile •Unknown durability

#### Accelerated cell manufacturing

In response to these challenges in CAR-T cell manufacturing, new strategies have been developed to dramatically decrease the duration of cell manufacturing, thereby reducing production costs, probability of manufacturing failures, and vein-to-vein time (Barba et al., 2022; Ghassemi et al., 2022; Sperling et al., 2023; Yang et al., 2022). These next-generation manufacturing processes can arrive at final-product formulation within as few as 24 h (Fig. 1), offering the possibility of significantly accelerating patient access to therapy. The bulk of the time-saving results from significant shortening of the ex vivo expansion period, thus limiting the fold increase in cell count that could be accomplished. However, the reduced number of ex vivo cell divisions could also result in a less differentiated T-cell population, with greater long-term proliferative potential after infusion. Indeed, early results indicate that products harvested at earlier time points exhibit greater proportions of T_CM_ cells (CD45RO^+^CD62L^+^) and T_N_/T_SCM_ cells (CD45RO^−^/CCR7^+^), which have been reported to exhibit therapeutically favorable phenotype and function (Flinn et al., 2021; Ghassemi et al., 2022; Yang et al., 2022).

One example of a CAR-T cell product made with accelerated manufacturing is the CD19-directed YTB323 cells manufactured with the “T-Charge” platform, which requires <2 d in ex vivo culture. YTB323 cells have been reported to exhibit an enrichment of T_N_ and T_SCM_ cells, as well as a more similar CD4:CD8 ratio as that found in the apheresis material, in comparison to the equivalent product produced using the conventional CAR-T cell manufacturing process (tisa-cel; Flinn et al., 2021). Of note, preliminary results from a phase-2 trial indicate that YTB323 can yield comparable efficacy in patients with relapsed/refractory diffuse large B-cell lymphoma (DLBCL) as tisa-cel but at a 25-fold lower dose, suggesting the reduced ex vivo culture time may have yielded a functionally superior product (Dickinson et al., 2023). As another example, the BCMA-directed PHE885 cells manufactured with the same T-Charge platform achieved a 98% overall response rate in a phase-1 trial for patients with relapsed/refractory MM (Sperling et al., 2023). Of note, CAR-T cells were detectible at 6 mo after infusion in 93% (13/14) of patients and at 12 mo in 71% (5/7) of patients, indicating robust in vivo T-cell persistence (Sperling et al., 2023).

Another accelerated manufacturing platform, termed FasT CAR-T, has been used to generate “CD19 F-CAR-T” cells for patients with B-ALL, with 92% (23/25) of patients achieving minimum residual disease-negative complete responses (Yang et al., 2022). The majority (20/25) of patients in this trial proceeded to undergo allogeneic hematopoietic stem-cell transplantation, and thus the long-term durability of response to CAR-T cell therapy could not be assessed. As the clinical data from multiple trials continue to mature, the relative durability of response induced by CAR-T cell products generated through different manufacturing platforms will be a significant point of interest.

A theoretical concern associated with the accelerated cell manufacturing process is that CAR-T cells cryopreserved shortly after ex vivo activation may exhibit an overly stimulated phenotype. Furthermore, particularly in the context of hematological malignancies, products from highly abbreviated manufacturing processes have increased probability of containing tumor cells that would otherwise have been depleted during a prolonged ex vivo culture period under conditions optimized for T-cell growth. However, available clinical data suggest that the safety profile of CAR-T cell products manufactured through these shortened protocols remains clinically manageable. Phase 1 study results for YTB323 in the treatment of DLBCL showed 33% (15/45) of patients experienced CRS, with one grade-4 event, and 11% (5/45) experienced immune effector cell-associated neurotoxicity syndrome (ICANS), with two grade-3 events (Flinn et al., 2021). In patients with B-ALL, CD19 F-CAR-T cells exhibited greater toxicity, tripling the incidence of CRS (96% overall; 24% grade ≥3) and doubling ICANS (28%, all grade ≥3). Twenty-one of 25 patients required interventional corticosteroids during the CRS onset period, though treatment did not ablate peak CAR-T cell expansion (Yang et al., 2022). Additional data from ongoing clinical trials will be highly informative in establishing our understanding of the biological differences among CAR-T cell products manufactured through different platforms. Finally, it is important to note that regulatory requirements on product release testing remain applicable to products manufactured with accelerated processes, and the time required for such release testing still needs to be accounted for in estimating the vein-to-vein time.

#### Process automation

Another avenue by which commercial CAR-T cell production may be improved involves the automation of the manufacturing process, which has the potential to reduce production costs, the probability of manufacturing failures attributed to human error, and contamination via touchpoints (Aleksandrova et al., 2019; Mock et al., 2016; Trainor et al., 2023). In certain jurisdictions (e.g., the United States), some automated systems are considered fully enclosed and allowed to be operated outside facilities that meet Good Manufacturing Practice standards. Furthermore, automation could significantly reduce the number and experience level required for the manufacturing staff (Zhu et al., 2018). These factors, in turn, support the possibility of on-site manufacturing that provides fresh, non-cryopreserved products for patients. In a phase-1 trial for patients with non-Hodgkin lymphoma, patients were given CD19/CD20 bispecific CAR-T cells that were either administered fresh or thawed from cryopreserved aliquots, with early data showing fresh CAR-T cell products had higher viability (93% fresh versus 63% cryopreserved; Shah et al., 2019b). A follow-up report on the trial confirmed that patients treated with fresh CAR-T cell products experienced substantially higher complete response rates (80% fresh versus 29% cryopreserved) as well as greater CAR-T cell expansion and persistence compared with patients treated with cryopreserved CAR-T cells (Shah et al., 2020). Although it remains possible that improvements in cell-cryopreservation procedure could reduce the difference in efficacy observed between fresh and cryopreserved CAR-T cell products, these results highlight the potential advantage of on-site cell manufacturing. However, scaling such a practice to a large number of medical centers while ensuring consistent product quality remains a key challenge. Importantly, several regulatory changes would be required to allow point-of-care CAR-T cell manufacturing, and differences among jurisdictions across the globe represent substantial hurdles in the practical implementation of on-site cell manufacturing (Elsallab and Maus, 2023). Furthermore, the use of fresh cell products precludes the completion of release testing that requires long incubation periods, necessitating reliance on in-process sample testing as well as contingency clinical management plans for scenarios in which failed sterility or other test results are received after the cell product has already been infused. In addition, the administration of fresh products poses significant logistical challenges as it requires close temporal coordination between cell manufacturing and patient readiness for receiving cell infusion. Finally, the highly standardized nature of automated systems can pose challenges when the optimal process-parameter setting needs to be adjusted on a patient-by-patient basis. For example, cells from different patients could have dramatically different expansion rates, thus the amount of media needed on each day to maintain the proper cell density in the culture could vary widely across manufacturing campaigns. To achieve the optimal outcome, instruments need to be equipped to sense critical parameters and respond accordingly.

#### In vivo cell manufacturing

Looking further ahead, the next-next-generation manufacturing process may completely eliminate ex vivo cell manipulation and expansion, and instead produce therapeutic cell populations in vivo (Michels et al., 2022). Several preclinical studies have demonstrated the ability to transduce T cells in vivo using lentivirus or adeno-associated virus (Agarwal et al., 2020; Frank et al., 2020; Huckaby et al., 2021; Michels et al., 2023; Pfeiffer et al., 2018). Furthermore, lipid nanoparticles (LNPs) and polymeric nanocarriers have been shown to deliver nucleic-acid payloads to T cells in vivo (Rurik et al., 2022; Smith et al., 2017). To achieve T-cell targeting, the viral particles, LNPs, and nanocarriers are typically decorated with a single-chain variable fragment or other binding domains targeted to CD3, CD4, or CD8 (Michels et al., 2022). However, the specificity for T-cell targeting may not need to be absolute, and one could contemplate the potential advantage of simultaneously generating CAR-T cells, CAR–natural killer cells, and CAR-macrophages. Nevertheless, it remains critical that the transgene is not delivered into malignant cells, as such an integration event could result in CAR proteins masking the target antigen and shielding the tumor cell from detection by CAR-expressing effector cells (Ruella et al., 2018). Furthermore, off-target gene delivery increases the risk of genotoxicity in the case of viral vectors that can achieve stable transgene integration. In practical terms, specifically targeting the transgene-delivery vehicle to T cells and strategies to facilitate efficient transgene integration without external T-cell activation are likely necessary to generate an effective dose of CAR-T cells that can achieve durable antitumor efficacy. Finally, in-depth pharmacokinetics and safety demonstrations will be needed prior to clinical translation of in vivo CAR-T cell manufacturing platforms. Despite the many hurdles that remain, the ability to bypass ex vivo cell manufacturing has the potential to significantly reduce cost and increase access to CAR-T cell therapy for patients in need of this treatment option.

## Concluding remarks

CAR-T cell therapy has become an increasingly important treatment option for hematological malignancies, and clinical trials continue to expand CAR-T cell therapy’s application to the treatment of solid tumors and autoimmune diseases. Consequently, CAR-T cell manufacturing processes that can efficiently and reliably produce high-quality cell products have become essential for supporting timely, safe, and efficacious patient care. Accumulating clinical experience, real-world manufacturing data collection, advancements in automated system engineering, and fundamental understanding of T-cell biology all play critical roles in the continual improvement of autologous CAR-T cell manufacturing processes. Furthermore, the rapidly evolving landscape of allogeneic cell therapy involving additional immune effector cell types as well as T cells generated from stem-cell populations will continue to be a source of both intriguing scientific questions and practical engineering challenges in the coming years.

## References

[bib1] Abramson, J.S., M.L. Palomba, L.I. Gordon, M.A. Lunning, M. Wang, J. Arnason, A. Mehta, E. Purev, D.G. Maloney, C. Andreadis, . 2020. Lisocabtagene maraleucel for patients with relapsed or refractory large B-cell lymphomas (TRANSCEND NHL 001): A multicentre seamless design study. Lancet. 396:839–852. 10.1016/S0140-6736(20)31366-0 32888407

[bib2] Abramson, J.S., S.R. Solomon, J. Arnason, P.B. Johnston, B. Glass, V. Bachanova, S. Ibrahimi, S. Mielke, P. Mutsaers, F. Hernandez-Ilizaliturri, . 2023. Lisocabtagene maraleucel as second-line therapy for large B-cell lymphoma: Primary analysis of the phase 3 TRANSFORM study. Blood. 141:1675–1684. 10.1182/blood.2022018730 36542826 PMC10646768

[bib3] Agarwal, S., J.D.S. Hanauer, A.M. Frank, V. Riechert, F.B. Thalheimer, and C.J. Buchholz. 2020. In vivo generation of CAR T cells selectively in human CD4^+^ lymphocytes. Mol. Ther. 28:1783–1794. 10.1016/j.ymthe.2020.05.005 32485137 PMC7403353

[bib4] Al Hadidi, S., A. Szabo, J. Esselmann, L. Hammons, M. Hussain, Y. Ogunsesan, N. Thalambedu, F. Khan, J. Sethi, A. Janardan, . 2023. Clinical outcome of patients with relapsed refractory multiple myeloma listed for BCMA directed commercial CAR-T therapy. Bone Marrow Transpl. 58:443–445. 10.1038/s41409-022-01905-1 36550200

[bib5] Aleksandrova, K., J. Leise, C. Priesner, A. Melk, F. Kubaink, H. Abken, A. Hombach, M. Aktas, M. Essl, I. Bürger, . 2019. Functionality and cell senescence of CD4/CD8-selected CD20 CAR T cells manufactured using the automated CliniMACS Prodigy® platform. Transfus. Med. Hemother. 46:47–54. 10.1159/000495772 31244581 PMC6558326

[bib6] Arcangeli, S., C. Bove, C. Mezzanotte, B. Camisa, L. Falcone, F. Manfredi, E. Bezzecchi, R. El Khoury, R. Norata, F. Sanvito, . 2022. CAR T cell manufacturing from naive/stem memory T lymphocytes enhances antitumor responses while curtailing cytokine release syndrome. J. Clin. Invest. 132:e150807. 10.1172/JCI150807 35503659 PMC9197529

[bib7] Arcangeli, S., L. Falcone, B. Camisa, F. De Girardi, M. Biondi, F. Giglio, F. Ciceri, C. Bonini, A. Bondanza, and M. Casucci. 2020. Next-generation manufacturing protocols enriching T_SCM_ CAR T cells can overcome disease-specific T cell defects in cancer patients. Front. Immunol. 11:1217. 10.3389/fimmu.2020.01217 32636841 PMC7317024

[bib8] Bai, Z., S. Woodhouse, Z. Zhao, R. Arya, K. Govek, D. Kim, S. Lundh, A. Baysoy, H. Sun, Y. Deng, . 2022. Single-cell antigen-specific landscape of CAR T infusion product identifies determinants of CD19-positive relapse in patients with ALL. Sci. Adv. 8:eabj2820. 10.1126/sciadv.abj2820 35675405 PMC9177075

[bib9] Balke-Want, H., V. Keerthi, A. Cadinanos-Garai, C. Fowler, N. Gkitsas, A.K. Brown, R. Tunuguntla, M. Abou-El-Enein, and S.A. Feldman. 2023. Non-viral chimeric antigen receptor (CAR) T cells going viral. Immunooncol. Technol. 18:100375. 10.1016/j.iotech.2023.100375 37124148 PMC10139995

[bib10] Barba, P., M. Kwon, J. Briones, U. Jaeger, E. Bachy, D. Blaise, N. Boissel, K. Kato, N.N. Shah, M.J. Frigault, . 2022. YTB323 (rapcabtagene autoleucel) demonstrates durable efficacy and a manageable safety profile in patients with relapsed/refractory diffuse large B-cell lymphoma: Phase I study update. Blood. 140:1056–1059. 10.1182/blood-2022-16252036074532

[bib11] Beatty, G.L., A.R. Haas, M.V. Maus, D.A. Torigian, M.C. Soulen, G. Plesa, A. Chew, Y. Zhao, B.L. Levine, S.M. Albelda, . 2014. Mesothelin-specific chimeric antigen receptor mRNA-engineered T cells induce anti-tumor activity in solid malignancies. Cancer Immunol. Res. 2:112–120. 10.1158/2326-6066.CIR-13-0170 24579088 PMC3932715

[bib12] Beider, K., M.J. Besser, J. Schachter, A.H. Grushchenko-Polaq, V. Voevoda, I. Wolf, O. Ostrovsky, E. Jacoby, A. Shimoni, and A. Nagler. 2019. Upregulation of senescent/exhausted phenotype of CAR T cells and induction of both Treg and myeloid suppressive cells correlate with reduced response to CAR T cell therapy in relapsed/refractory B cell malignancies. Blood. 134:3234. 10.1182/blood-2019-128068

[bib13] Berdeja, J.G., D. Madduri, S.Z. Usmani, A. Jakubowiak, M. Agha, A.D. Cohen, A.K. Stewart, P. Hari, M. Htut, A. Lesokhin, . 2021. Ciltacabtagene autoleucel, a B-cell maturation antigen-directed chimeric antigen receptor T-cell therapy in patients with relapsed or refractory multiple myeloma (CARTITUDE-1): A phase 1b/2 open-label study. Lancet. 398:314–324. 10.1016/S0140-6736(21)00933-8 34175021

[bib14] Berger, C., M.C. Jensen, P.M. Lansdorp, M. Gough, C. Elliott, and S.R. Riddell. 2008. Adoptive transfer of effector CD8+ T cells derived from central memory cells establishes persistent T cell memory in primates. J. Clin. Invest. 118:294–305. 10.1172/JCI32103 18060041 PMC2104476

[bib15] Bhaskar, S.T., B.R. Dholaria, S.M. Sengsayadeth, B.N. Savani, and O.O. Oluwole. 2021. Role of bridging therapy during chimeric antigen receptor T cell therapy. eJHaem. 3:39–45. 10.1002/jha2.335 35844303 PMC9175845

[bib16] Bishop, D.C., L.E. Clancy, R. Simms, J. Burgess, G. Mathew, L. Moezzi, J.A. Street, G. Sutrave, E. Atkins, H.M. McGuire, . 2021. Development of CAR T-cell lymphoma in 2 of 10 patients effectively treated with piggyBac-modified CD19 CAR T cells. Blood. 138:1504–1509. 10.1182/blood.2021010813 34010392

[bib17] Bos, R., and L.A. Sherman. 2010. CD4+ T-cell help in the tumor milieu is required for recruitment and cytolytic function of CD8+ T lymphocytes. Cancer Res. 70:8368–8377. 10.1158/0008-5472.CAN-10-1322 20940398 PMC2970736

[bib18] Cappell, K.M., and J.N. Kochenderfer. 2023. Long-term outcomes following CAR T cell therapy: What we know so far. Nat. Rev. Clin. Oncol. 20:359–371. 10.1038/s41571-023-00754-1 37055515 PMC10100620

[bib19] Casati, A., A. Varghaei-Nahvi, S.A. Feldman, M. Assenmacher, S.A. Rosenberg, M.E. Dudley, and A. Scheffold. 2013. Clinical-scale selection and viral transduction of human naïve and central memory CD8+ T cells for adoptive cell therapy of cancer patients. Cancer Immunol. Immunother. 62:1563–1573. 10.1007/s00262-013-1459-x 23903715 PMC6348480

[bib20] Castellino, F., and R.N. Germain. 2006. Cooperation between CD4+ and CD8+ T cells: When, where, and how. Annu. Rev. Immunol. 24:519–540. 10.1146/annurev.immunol.23.021704.115825 16551258

[bib159] Center for Biologics Evaluation and Research. 2020. Testing of Retroviral Vector-Based Human Gene Therapy Products for Replication Competent Retrovirus during Product Manufacture and Patient Follow-Up. U.S. Food & Drug Administration. https://www.fda.gov/regulatory-information/search-fda-guidance-documents/testing-retroviral-vector-based-human-gene-therapy-products-replication-competent-retrovirus-during

[bib157] Center for Biologics Evaluation and Research. 2022a. Considerations for the Development of Chimeric Antigen Receptor (CAR) T Cell Products: Draft Guidance for Industry. U.S. Food & Drug Administration. https://www.fda.gov/regulatory-information/search-fda-guidance-documents/considerations-development-chimeric-antigen-receptor-car-t-cell-products

[bib158] Center for Biologics Evaluation and Research. 2022b. FDA CBER OTAT Town Hall: Gene Therapy Chemistry, Manufacturing, and Controls. U.S. Food & Drug Administration. https://www.fda.gov/news-events/otat-town-hall-gene-therapy-chemistry-manufacturing-and-controls-09292022

[bib24] Ceppi, F., A.L. Wilson, C. Annesley, G.R. Kimmerly, C. Summers, A. Brand, K. Seidel, Q.V. Wu, A. Beebe, C. Brown, . 2022. Modified manufacturing process modulates CD19CAR T-cell engraftment fitness and leukemia-free survival in pediatric and young adult subjects. Cancer Immunol. Res. 10:856–870. 10.1158/2326-6066.CIR-21-0501 35580141 PMC9250626

[bib25] Chang, J.T., E.J. Wherry, and A.W. Goldrath. 2014. Molecular regulation of effector and memory T cell differentiation. Nat. Immunol. 15:1104–1115. 10.1038/ni.3031 25396352 PMC4386685

[bib26] Chen, A.J., J. Zhang, A. Agarwal, and D.N. Lakdawalla. 2022. Value of reducing wait times for chimeric antigen receptor T-cell treatment: Evidence from randomized controlled trial data on tisagenlecleucel for diffuse large B-cell lymphoma. Value Health. 25:1344–1351. 10.1016/j.jval.2022.02.007 35341689

[bib27] Chen, G.M., C. Chen, R.K. Das, P. Gao, C.H. Chen, S. Bandyopadhyay, Y.Y. Ding, Y. Uzun, W. Yu, Q. Zhu, . 2021. Integrative bulk and single-cell profiling of premanufacture T-cell populations reveals factors mediating long-term persistence of CAR T-cell therapy. Cancer Discov. 11:2186–2199. 10.1158/2159-8290.CD-20-1677 33820778 PMC8419030

[bib28] Cheung, A.S., D.K.Y. Zhang, S.T. Koshy, and D.J. Mooney. 2018. Scaffolds that mimic antigen-presenting cells enable ex vivo expansion of primary T cells. Nat. Biotechnol. 36:160–169. 10.1038/nbt.4047 29334370 PMC5801009

[bib30] Chong, E.A., B.L. Levine, S.A. Grupp, M.M. Davis, D.L. Siegel, S.L. Maude, W.L. Gladney, N.V. Frey, D.L. Porter, W.T. Hwang, . 2019. CAR T cell viability release testing and clinical outcomes: Is there a lower limit? Blood. 134:1873–1875. 10.1182/blood.2019002258 31554634 PMC6872962

[bib31] Colombo, M.P., and S. Piconese. 2007. Regulatory-T-cell inhibition versus depletion: The right choice in cancer immunotherapy. Nat. Rev. Cancer. 7:880–887. 10.1038/nrc2250 17957190

[bib29] Committee for Medicinal Products for Human Use. 2022. Assessment Report: Carvykti (EMA/594558/2022). European Medicines Agency. https://www.ema.europa.eu/en/medicines/human/EPAR/carvykti

[bib32] Cornetta, K., and W.F. Anderson. 1989. Protamine sulfate as an effective alternative to polybrene in retroviral-mediated gene-transfer: Implications for human gene therapy. J. Virol. Methods. 23:187–194. 10.1016/0166-0934(89)90132-8 2786000

[bib33] Cornetta, K., L. Duffy, C.J. Turtle, M. Jensen, S. Forman, G. Binder-Scholl, T. Fry, A. Chew, D.G. Maloney, and C.H. June. 2018. Absence of replication-competent lentivirus in the clinic: Analysis of infused T cell products. Mol. Ther. 26:280–288. 10.1016/j.ymthe.2017.09.008 28970045 PMC5762981

[bib34] Delville, M., T. Soheili, F. Bellier, A. Durand, A. Denis, C. Lagresle-Peyrou, M. Cavazzana, I. Andre-Schmutz, and E. Six. 2018. A nontoxic transduction enhancer enables highly efficient lentiviral transduction of primary murine T cells and hematopoietic stem cells. Mol. Ther. Methods Clin. Dev. 10:341–347. 10.1016/j.omtm.2018.08.002 30191160 PMC6125771

[bib35] Deng, Q., G. Han, N. Puebla-Osorio, M.C.J. Ma, P. Strati, B. Chasen, E. Dai, M. Dang, N. Jain, H. Yang, . 2020. Characteristics of anti-CD19 CAR T cell infusion products associated with efficacy and toxicity in patients with large B cell lymphomas. Nat. Med. 26:1878–1887. 10.1038/s41591-020-1061-7 33020644 PMC8446909

[bib36] Depil, S., P. Duchateau, S.A. Grupp, G. Mufti, and L. Poirot. 2020. ‘Off-the-shelf’ allogeneic CAR T cells: Development and challenges. Nat. Rev. Drug Discov. 19:185–199. 10.1038/s41573-019-0051-2 31900462

[bib37] Dias, J., A. Cadiñanos-Garai, and C. Roddie. 2023. Release assays and potency assays for CAR T-cell interventions. Adv. Exp. Med. Biol. 1420:117–137. 10.1007/978-3-031-30040-0_8 37258787

[bib38] Dickinson, M.J., P. Barba, U. Jäger, N.N. Shah, D. Blaise, J. Briones, L. Shune, N. Boissel, A. Bondanza, L. Mariconti, . 2023. A novel autologous CAR-T therapy, YTB323, with preserved T-cell stemness shows enhanced CAR T-cell efficacy in preclinical and early clinical development. Cancer Discov. 13:1982–1997. 10.1158/2159-8290.CD-22-1276 37249512 PMC10481129

[bib39] Dimitri, A., F. Herbst, and J.A. Fraietta. 2022. Engineering the next-generation of CAR T-cells with CRISPR-Cas9 gene editing. Mol. Cancer. 21:78. 10.1186/s12943-022-01559-z 35303871 PMC8932053

[bib40] Doms, R.W. 2016. Basic concepts: A step-by-step guide to viral infection. In Viral Pathogenesis: From Basics to Systems Biology. M.G. Katze, M.J. Korth, G.L. Law, and N. Nathanson, editors. Academic Press, San Diego. 10.1016/B978-0-12-800964-2.00003-3

[bib41] Elsallab, M., and M.V. Maus. 2023. Expanding access to CAR T cell therapies through local manufacturing. Nat. Biotechnol. 41:1698–1708. 10.1038/s41587-023-01981-8 37884746

[bib42] Eyquem, J., J. Mansilla-Soto, T. Giavridis, S.J. van der Stegen, M. Hamieh, K.M. Cunanan, A. Odak, M. Gönen, and M. Sadelain. 2017. Targeting a CAR to the TRAC locus with CRISPR/Cas9 enhances tumour rejection. Nature. 543:113–117. 10.1038/nature21405 28225754 PMC5558614

[bib43] Farber, D.L., N.A. Yudanin, and N.P. Restifo. 2014. Human memory T cells: Generation, compartmentalization and homeostasis. Nat. Rev. Immunol. 14:24–35. 10.1038/nri3567 24336101 PMC4032067

[bib50] Finney, O.C., H.M. Brakke, S. Rawlings-Rhea, R. Hicks, D. Doolittle, M. Lopez, R.B. Futrell, R.J. Orentas, D. Li, R.A. Gardner, and M.C. Jensen. 2019. CD19 CAR T cell product and disease attributes predict leukemia remission durability. J. Clin. Invest. 129:2123–2132. 10.1172/JCI125423 30860496 PMC6486329

[bib51] Flinn, I.W., U. Jaeger, N. Shah, D. Blaise, J. Briones, L. Shune, N. Boissel, A. Bondanza, D. Lu, X. Zhu, . 2021. A first-in-human study of YTB323, a novel, autologous CD19-directed CAR-T cell therapy manufactured using the novel T-charge TM platform, for the treatment of patients (pts) with relapsed/refractory (r/r) diffuse large B-cell lymphoma (DLBCL). Blood. 138:740. 10.1182/blood-2021-146268

[bib52] Fowler, N.H., M. Dickinson, M. Dreyling, J. Martinez-Lopez, A. Kolstad, J. Butler, M. Ghosh, L. Popplewell, J.C. Chavez, E. Bachy, . 2022. Tisagenlecleucel in adult relapsed or refractory follicular lymphoma: The phase 2 ELARA trial. Nat. Med. 28:325–332. 10.1038/s41591-021-01622-0 34921238

[bib53] Foy, S.P., K. Jacoby, D.A. Bota, T. Hunter, Z. Pan, E. Stawiski, Y. Ma, W. Lu, S. Peng, C.L. Wang, . 2023. Non-viral precision T cell receptor replacement for personalized cell therapy. Nature. 615:687–696. 10.1038/s41586-022-05531-1 36356599 PMC9768791

[bib54] Fraietta, J.A., S.F. Lacey, E.J. Orlando, I. Pruteanu-Malinici, M. Gohil, S. Lundh, A.C. Boesteanu, Y. Wang, R.S. O’Connor, W.T. Hwang, . 2018a. Determinants of response and resistance to CD19 chimeric antigen receptor (CAR) T cell therapy of chronic lymphocytic leukemia. Nat. Med. 24:563–571. 10.1038/s41591-018-0010-1 29713085 PMC6117613

[bib55] Fraietta, J.A., C.L. Nobles, M.A. Sammons, S. Lundh, S.A. Carty, T.J. Reich, A.P. Cogdill, J.J.D. Morrissette, J.E. DeNizio, S. Reddy, . 2018b. Disruption of TET2 promotes the therapeutic efficacy of CD19-targeted T cells. Nature. 558:307–312. 10.1038/s41586-018-0178-z 29849141 PMC6320248

[bib56] Frank, A.M., A.H. Braun, L. Scheib, S. Agarwal, I.C. Schneider, F. Fusil, S. Perian, U. Sahin, F.B. Thalheimer, E. Verhoeyen, and C.J. Buchholz. 2020. Combining T-cell-specific activation and in vivo gene delivery through CD3-targeted lentiviral vectors. Blood Adv. 4:5702–5715. 10.1182/bloodadvances.2020002229 33216892 PMC7686896

[bib57] Gao, T.A., and Y.Y. Chen. 2022. Engineering next-generation CAR-T cells: Overcoming tumor hypoxia and metabolism. Annu. Rev. Chem. Biomol. Eng. 13:193–216. 10.1146/annurev-chembioeng-092120-092914 35700528

[bib58] Ghassemi, S., J.S. Durgin, S. Nunez-Cruz, J. Patel, J. Leferovich, M. Pinzone, F. Shen, K.D. Cummins, G. Plesa, V.A. Cantu, . 2022. Rapid manufacturing of non-activated potent CAR T cells. Nat. Biomed. Eng. 6:118–128. 10.1038/s41551-021-00842-6 35190680 PMC8860360

[bib59] Gogol-Döring, A., I. Ammar, S. Gupta, M. Bunse, C. Miskey, W. Chen, W. Uckert, T.F. Schulz, Z. Izsvák, and Z. Ivics. 2016. Genome-wide profiling reveals remarkable parallels between insertion site selection properties of the MLV retrovirus and the piggyBac transposon in primary human CD4(+) T cells. Mol. Ther. 24:592–606. 10.1038/mt.2016.11 26755332 PMC4786924

[bib60] Golubovskaya, V., and L. Wu. 2016. Different subsets of T cells, memory, effector functions, and CAR-T immunotherapy. Cancers. 8:36. 10.3390/cancers8030036 26999211 PMC4810120

[bib61] Good, Z., J.Y. Spiegel, B. Sahaf, M.B. Malipatlolla, Z.J. Ehlinger, S. Kurra, M.H. Desai, W.D. Reynolds, A. Wong Lin, P. Vandris, . 2022. Post-infusion CAR T_Reg_ cells identify patients resistant to CD19-CAR therapy. Nat. Med. 28:1860–1871. 10.1038/s41591-022-01960-7 36097223 PMC10917089

[bib62] Hamieh, M., J. Mansilla-Soto, I. Rivière, and M. Sadelain. 2023. Programming CAR T cell tumor recognition: Tuned antigen sensing and logic gating. Cancer Discov. 13:829–843. 10.1158/2159-8290.CD-23-0101 36961206 PMC10068450

[bib63] Hansen, D.K., S. Sidana, L.C. Peres, C. Colin Leitzinger, L. Shune, A. Shrewsbury, R. Gonzalez, D.W. Sborov, C. Wagner, D. Dima, . 2023. Idecabtagene vicleucel for relapsed/refractory multiple myeloma: Real-world experience from the myeloma CAR T consortium. J. Clin. Oncol. 41:2087–2097. 10.1200/JCO.22.01365 36623248 PMC10082273

[bib64] Haradhvala, N.J., M.B. Leick, K. Maurer, S.H. Gohil, R.C. Larson, N. Yao, K.M.E. Gallagher, K. Katsis, M.J. Frigault, J. Southard, . 2022. Distinct cellular dynamics associated with response to CAR-T therapy for refractory B cell lymphoma. Nat. Med. 28:1848–1859. 10.1038/s41591-022-01959-0 36097221 PMC9509487

[bib162] Havert, M. 2017. Summary Basis for Regulatory Action: axicabtagene ciloleucel. U.S. Food & Drug Administration. https://www.fda.gov/files/vaccines%2C%20blood%20%26%20biologics/published/October-18--2017-Summary-Basis-for-Regulatory-Action---YESCARTA.pdf

[bib65] Hou, A.J., L.C. Chen, and Y.Y. Chen. 2021. Navigating CAR-T cells through the solid-tumour microenvironment. Nat. Rev. Drug Discov. 20:531–550. 10.1038/s41573-021-00189-2 33972771

[bib66] Huckaby, J.T., E. Landoni, T.M. Jacobs, B. Savoldo, G. Dotti, and S.K. Lai. 2021. Bispecific binder redirected lentiviral vector enables in vivo engineering of CAR-T cells. J. Immunother. Cancer. 9:e002737. 10.1136/jitc-2021-002737 34518288 PMC8438880

[bib67] Jacobson, C.A., J.C. Chavez, A.R. Sehgal, B.M. William, J. Munoz, G. Salles, P.N. Munshi, C. Casulo, D.G. Maloney, S. de Vos, . 2022. Axicabtagene ciloleucel in relapsed or refractory indolent non-hodgkin lymphoma (ZUMA-5): A single-arm, multicentre, phase 2 trial. Lancet Oncol. 23:91–103. 10.1016/S1470-2045(21)00591-X 34895487

[bib68] Jacobson, C.A., F.L. Locke, D.B. Miklos, J.M. Vose, Y. Lin, L.E. Budde, D.G. Maloney, S. Jaglowski, P.A. Riedell, L.J. Lekakis, . 2020. Outcomes of patients (pts) in ZUMA-9, a multicenter, open-label study of axicabtagene ciloleucel (Axi-Cel) in relapsed/refractory large B cell lymphoma (R/R LBCL) for expanded access and commercial out-of-specification (OOS). Blood. 136:2–3. 10.1182/blood-2020-136136

[bib69] Jagannath, S., Y. Lin, H. Goldschmidt, D. Reece, A. Nooka, A. Senin, P. Rodriguez-Otero, R. Powles, K. Matsue, N. Shah, . 2021. KarMMa-RW: Comparison of idecabtagene vicleucel with real-world outcomes in relapsed and refractory multiple myeloma. Blood Cancer J. 11:116. 10.1038/s41408-021-00507-2 34145225 PMC8213772

[bib70] Jain, N., Z. Zhao, J. Feucht, R. Koche, A. Iyer, A. Dobrin, J. Mansilla-Soto, J. Yang, Y. Zhan, M. Lopez, . 2023. TET2 guards against unchecked BATF3-induced CAR T cell expansion. Nature. 615:315–322. 10.1038/s41586-022-05692-z 36755094 PMC10511001

[bib71] Jiang, F., and J.A. Doudna. 2017. CRISPR-Cas9 structures and mechanisms. Annu. Rev. Biophys. 46:505–529. 10.1146/annurev-biophys-062215-010822 28375731

[bib72] Jing, R., I. Scarfo, M.A. Najia, E. Lummertz da Rocha, A. Han, M. Sanborn, T. Bingham, C. Kubaczka, D.K. Jha, M. Falchetti, . 2022. EZH1 repression generates mature iPSC-derived CAR T cells with enhanced antitumor activity. Cell Stem Cell. 29:1181–1196.e6. 10.1016/j.stem.2022.06.014 35931029 PMC9386785

[bib73] Jo, T., S. Yoshihara, Y. Okuyama, K. Fujii, T. Henzan, K. Kahata, R. Yamazaki, W. Takeda, Y. Umezawa, K. Fukushima, . 2023. Risk factors for CAR-T cell manufacturing failure among DLBCL patients: A nationwide survey in Japan. Br. J. Haematol. 202:256–266. 10.1111/bjh.18831 37096915

[bib74] Kamdar, M., S.R. Solomon, J. Arnason, P.B. Johnston, B. Glass, V. Bachanova, S. Ibrahimi, S. Mielke, P. Mutsaers, F. Hernandez-Ilizaliturri, . 2022. Lisocabtagene maraleucel versus standard of care with salvage chemotherapy followed by autologous stem cell transplantation as second-line treatment in patients with relapsed or refractory large B-cell lymphoma (TRANSFORM): Results from an interim analysis of an open-label, randomised, phase 3 trial. Lancet. 399:2294–2308. 10.1016/S0140-6736(22)00662-6 35717989

[bib75] Kishton, R.J., M. Sukumar, and N.P. Restifo. 2017. Metabolic regulation of T cell longevity and function in tumor immunotherapy. Cell Metab. 26:94–109. 10.1016/j.cmet.2017.06.016 28683298 PMC5543711

[bib76] Kourelis, T., R. Bansal, J. Berdeja, D. Siegel, K. Patel, S. Mailankody, M. Htut, N. Shah, S.W. Wong, S. Sidana, . 2023. Ethical challenges with multiple myeloma BCMA chimeric antigen receptor T cell slot allocation: A multi-institution experience. Transpl. Cell. Ther. 29:255–258. 10.1016/j.jtct.2023.01.012 PMC10040426 36681151

[bib77] Kumamoto, Y., L.M. Mattei, S. Sellers, G.W. Payne, and A. Iwasaki. 2011. CD4+ T cells support cytotoxic T lymphocyte priming by controlling lymph node input. Proc. Natl. Acad. Sci. USA. 108:8749–8754. 10.1073/pnas.1100567108 21555577 PMC3102372

[bib78] Künkele, A., C. Brown, A. Beebe, S. Mgebroff, A.J. Johnson, A. Taraseviciute, L.S. Rolczynski, C.A. Chang, O.C. Finney, J.R. Park, and M.C. Jensen. 2019. Manufacture of chimeric antigen receptor T cells from mobilized cyropreserved peripheral blood stem cell units depends on monocyte depletion. Biol. Blood Marrow Transpl. 25:223–232. 10.1016/j.bbmt.2018.10.004 30315942

[bib164] Kwilas, A. 2021. Summary Basis for Regulatory Action: idecabtagene vicleucel. https://fda.report/media/147627/Summary+Basis+for+Regulatory+Action+-+ABECMA.pdf

[bib79] Labanieh, L., and C.L. Mackall. 2023. CAR immune cells: Design principles, resistance and the next generation. Nature. 614:635–648. 10.1038/s41586-023-05707-3 36813894

[bib80] Labbé, R.P., S. Vessillier, and Q.A. Rafiq. 2021. Lentiviral vectors for T cell engineering: Clinical applications, bioprocessing and future perspectives. Viruses. 13:1528. 10.3390/v13081528 34452392 PMC8402758

[bib81] Larson, S.M., C.M. Walthers, B. Ji, S.N. Ghafouri, J. Naparstek, J. Trent, J.M. Chen, M. Roshandell, C. Harris, M. Khericha, . 2023. CD19/CD20 bispecific chimeric antigen receptor (CAR) in naive/memory T cells for the treatment of relapsed or refractory non-hodgkin lymphoma. Cancer Discov. 13:580–597. 10.1158/2159-8290.CD-22-0964 36416874 PMC9992104

[bib82] Lee, D.H., F. Cervantes-Contreras, S.Y. Lee, D.J. Green, and B.G. Till. 2018. Improved expansion and function of CAR T cell products from cultures initiated at defined CD4:CD8 ratios. Blood. 132:3334. 10.1182/blood-2018-99-111576

[bib83] Levine, B.L., J. Miskin, K. Wonnacott, and C. Keir. 2016. Global manufacturing of CAR T cell therapy. Mol. Ther. Methods Clin. Dev. 4:92–101. 10.1016/j.omtm.2016.12.006 28344995 PMC5363291

[bib163] Liu, X.V. 2017. Summary Basis for Regulatory Action: tisagenlecleucel. U.S. Food & Drug Administration. https://www.fda.gov/files/vaccines%2C%20blood%20%26%20biologics/published/August-30--2017-Summary-Basis-for-Regulatory-Action---KYMRIAH.pdf

[bib84] Locke, F.L., D.B. Miklos, C.A. Jacobson, M.A. Perales, M.J. Kersten, O.O. Oluwole, A. Ghobadi, A.P. Rapoport, J. McGuirk, J.M. Pagel, . 2022. Axicabtagene ciloleucel as second-line therapy for large B-cell lymphoma. N. Engl. J. Med. 386:640–654. 10.1056/NEJMoa2116133 34891224

[bib85] Long, A.H., W.M. Haso, J.F. Shern, K.M. Wanhainen, M. Murgai, M. Ingaramo, J.P. Smith, A.J. Walker, M.E. Kohler, V.R. Venkateshwara, . 2015. 4-1BB costimulation ameliorates T cell exhaustion induced by tonic signaling of chimeric antigen receptors. Nat. Med. 21:581–590. 10.1038/nm.3838 25939063 PMC4458184

[bib86] Lyon, D., N. Lapteva, and A.P. Gee. 2018. Absence of replication-competent retrovirus in vectors, T cell products, and patient follow-up samples. Mol. Ther. 26:6–7. 10.1016/j.ymthe.2017.12.003 29301109 PMC5763145

[bib87] Mackensen, A., F. Müller, D. Mougiakakos, S. Böltz, A. Wilhelm, M. Aigner, S. Völkl, D. Simon, A. Kleyer, L. Munoz, . 2022. Anti-CD19 CAR T cell therapy for refractory systemic lupus erythematosus. Nat. Med. 28:2124–2132. 10.1038/s41591-022-02017-5 36109639

[bib88] Majzner, R.G., S. Ramakrishna, K.W. Yeom, S. Patel, H. Chinnasamy, L.M. Schultz, R.M. Richards, L. Jiang, V. Barsan, R. Mancusi, . 2022. GD2-CAR T cell therapy for H3K27M-mutated diffuse midline gliomas. Nature. 603:934–941. 10.1038/s41586-022-04489-4 35130560 PMC8967714

[bib89] Marcucci, K.T., J.K. Jadlowsky, W.T. Hwang, M. Suhoski-Davis, V.E. Gonzalez, I. Kulikovskaya, M. Gupta, S.F. Lacey, G. Plesa, A. Chew, . 2018. Retroviral and lentiviral safety analysis of gene-modified T cell products and infused HIV and oncology patients. Mol. Ther. 26:269–279. 10.1016/j.ymthe.2017.10.012 29203150 PMC5763152

[bib90] Mateos, M.V., K. Weisel, T. Martin, J.G. Berdeja, A. Jakubowiak, A.K. Stewart, S. Jagannath, Y. Lin, J. Diels, F. Ghilotti, . 2023. Adjusted comparison of outcomes between patients from CARTITUDE-1 versus multiple myeloma patients with prior exposure to proteasome inhibitors, immunomodulatory drugs and anti-CD38 antibody from the prospective, multinational LocoMMotion study of real-world clinical practice. Haematologica. 108:2192–2204. 10.3324/haematol.2022.280482 36546453 PMC10388260

[bib91] Maude, S.L., T.W. Laetsch, J. Buechner, S. Rives, M. Boyer, H. Bittencourt, P. Bader, M.R. Verneris, H.E. Stefanski, G.D. Myers, . 2018. Tisagenlecleucel in children and young adults with B-cell lymphoblastic leukemia. N. Engl. J. Med. 378:439–448. 10.1056/NEJMoa1709866 29385370 PMC5996391

[bib92] Melenhorst, J.J., G.M. Chen, M. Wang, D.L. Porter, C. Chen, M.A. Collins, P. Gao, S. Bandyopadhyay, H. Sun, Z. Zhao, . 2022. Decade-long leukaemia remissions with persistence of CD4^+^ CAR T cells. Nature. 602:503–509. 10.1038/s41586-021-04390-6 35110735 PMC9166916

[bib93] Mescher, M.F., J.M. Curtsinger, P. Agarwal, K.A. Casey, M. Gerner, C.D. Hammerbeck, F. Popescu, and Z. Xiao. 2006. Signals required for programming effector and memory development by CD8+ T cells. Immunol. Rev. 211:81–92. 10.1111/j.0105-2896.2006.00382.x 16824119

[bib94] Mian, A., and B.T. Hill. 2021. Brexucabtagene autoleucel for the treatment of relapsed/refractory mantle cell lymphoma. Expert Opin. Biol. Ther. 21:435–441. 10.1080/14712598.2021.1889510 33566715

[bib95] Michels, A., N. Ho, and C.J. Buchholz. 2022. Precision medicine: In vivo CAR therapy as a showcase for receptor-targeted vector platforms. Mol. Ther. 30:2401–2415. 10.1016/j.ymthe.2022.05.018 35598048 PMC9263322

[bib96] Michels, K.R., A. Sheih, S.A. Hernandez, A.H. Brandes, D. Parrilla, B. Irwin, A.M. Perez, H.A. Ting, C.J. Nicolai, T. Gervascio, . 2023. Preclinical proof of concept for VivoVec, a lentiviral-based platform for in vivo CAR T-cell engineering. J. Immunother. Cancer. 11:e006292. 10.1136/jitc-2022-006292 36918221 PMC10016276

[bib97] Micklethwaite, K.P., K. Gowrishankar, B.S. Gloss, Z. Li, J.A. Street, L. Moezzi, M.A. Mach, G. Sutrave, L.E. Clancy, D.C. Bishop, . 2021. Investigation of product-derived lymphoma following infusion of piggyBac-modified CD19 chimeric antigen receptor T cells. Blood. 138:1391–1405. 10.1182/blood.2021010858 33974080 PMC8532197

[bib98] Mock, U., L. Nickolay, B. Philip, G.W. Cheung, H. Zhan, I.C.D. Johnston, A.D. Kaiser, K. Peggs, M. Pule, A.J. Thrasher, and W. Qasim. 2016. Automated manufacturing of chimeric antigen receptor T cells for adoptive immunotherapy using CliniMACS prodigy. Cytotherapy. 18:1002–1011. 10.1016/j.jcyt.2016.05.009 27378344

[bib99] Monjezi, R., C. Miskey, T. Gogishvili, M. Schleef, M. Schmeer, H. Einsele, Z. Ivics, and M. Hudecek. 2017. Enhanced CAR T-cell engineering using non-viral Sleeping Beauty transposition from minicircle vectors. Leukemia. 31:186–194. 10.1038/leu.2016.180 27491640

[bib100] O’Rourke, D.M., M.P. Nasrallah, A. Desai, J.J. Melenhorst, K. Mansfield, J.J.D. Morrissette, M. Martinez-Lage, S. Brem, E. Maloney, A. Shen, . 2017. A single dose of peripherally infused EGFRvIII-directed CAR T cells mediates antigen loss and induces adaptive resistance in patients with recurrent glioblastoma. Sci. Transl. Med. 9:eaaa0984. 10.1126/scitranslmed.aaa0984 28724573 PMC5762203

[bib101] Odak, A., H. Yuan, J. Feucht, V.A. Cantu, J. Mansilla-Soto, F. Kogel, J. Eyquem, J. Everett, F.D. Bushman, C.S. Leslie, and M. Sadelain. 2023. Novel extragenic genomic safe harbors for precise therapeutic T-cell engineering. Blood. 141:2698–2712. 10.1182/blood.2022018924 36745870 PMC10273162

[bib102] Pfeiffer, A., F.B. Thalheimer, S. Hartmann, A.M. Frank, R.R. Bender, S. Danisch, C. Costa, W.S. Wels, U. Modlich, R. Stripecke, . 2018. In vivo Generation of human CD19-CAR T cells results in B-cell depletion and signs of cytokine release syndrome. EMBO Mol. Med. 10:e9158. 10.15252/emmm.201809158 30224381 PMC6220327

[bib160] Price, G. 2020. Summary Basis for Regulatory Action: Brexucabtagene Autoleucel. U.S. Food & Drug Administration. https://www.fda.gov/media/141093/download

[bib103] Prommersberger, S., M. Reiser, J. Beckmann, S. Danhof, M. Amberger, P. Quade-Lyssy, H. Einsele, M. Hudecek, H. Bonig, and Z. Ivics. 2021. CARAMBA: A first-in-human clinical trial with SLAMF7 CAR-T cells prepared by virus-free sleeping beauty gene transfer to treat multiple myeloma. Gene Ther. 28:560–571. 10.1038/s41434-021-00254-w 33846552 PMC8455317

[bib104] Puig-Saus, C., B. Sennino, S. Peng, C.L. Wang, Z. Pan, B. Yuen, B. Purandare, D. An, B.B. Quach, D. Nguyen, . 2023. Neoantigen-targeted CD8^+^ T cell responses with PD-1 blockade therapy. Nature. 615:697–704. 10.1038/s41586-023-05787-1 36890230 PMC10441586

[bib105] Rajabzadeh, A., A.A. Hamidieh, and F. Rahbarizadeh. 2021. Spinoculation and retronectin highly enhance the gene transduction efficiency of Mucin-1-specific chimeric antigen receptor (CAR) in human primary T cells. BMC Mol. Cell Biol. 22:57. 10.1186/s12860-021-00397-z 34814824 PMC8609792

[bib106] Raje, N., J. Berdeja, Y. Lin, D. Siegel, S. Jagannath, D. Madduri, M. Liedtke, J. Rosenblatt, M.V. Maus, A. Turka, . 2019. Anti-BCMA CAR T-cell therapy bb2121 in relapsed or refractory multiple myeloma. N. Engl. J. Med. 380:1726–1737. 10.1056/NEJMoa1817226 31042825 PMC8202968

[bib107] Roberts, Z.J., M. Better, A. Bot, M.R. Roberts, and A. Ribas. 2018. Axicabtagene ciloleucel, a first-in-class CAR T cell therapy for aggressive NHL. Leuk. Lymphoma. 59:1785–1796. 10.1080/10428194.2017.1387905 29058502

[bib108] Roddie, C., L. Neill, W. Osborne, S. Iyengar, E. Tholouli, D. Irvine, S. Chaganti, C. Besley, A. Bloor, C. Jones, . 2023. Effective bridging therapy can improve CD19 CAR-T outcomes while maintaining safety in patients with large B-cell lymphoma. Blood Adv. 7:2872–2883. 10.1182/bloodadvances.2022009019 36724512 PMC10300297

[bib109] Rossoff, J., C. Baggott, S. Prabhu, H. Pacenta, C. Phillips, H.E. Stefanski, J. Talano, A. Moskop, S.P. Margossian, M.R. Verneris, . 2020. Real-world treatment of pediatric patients with relapsed/refractory B-Cell acute lymphoblastic leukemia using tisagenlecleucel that is out of specification for commercial release. Blood. 136:42–44. 10.1182/blood-2020-136674

[bib110] Ruella, M., J. Xu, D.M. Barrett, J.A. Fraietta, T.J. Reich, D.E. Ambrose, M. Klichinsky, O. Shestova, P.R. Patel, I. Kulikovskaya, . 2018. Induction of resistance to chimeric antigen receptor T cell therapy by transduction of a single leukemic B cell. Nat. Med. 24:1499–1503. 10.1038/s41591-018-0201-9 30275568 PMC6511988

[bib111] Rurik, J.G., I. Tombácz, A. Yadegari, P.O. Méndez Fernández, S.V. Shewale, L. Li, T. Kimura, O.Y. Soliman, T.E. Papp, Y.K. Tam, . 2022. CAR T cells produced in vivo to treat cardiac injury. Science. 375:91–96. 10.1126/science.abm0594 34990237 PMC9983611

[bib112] Sallusto, F., J. Geginat, and A. Lanzavecchia. 2004. Central memory and effector memory T cell subsets: Function, generation, and maintenance. Annu. Rev. Immunol. 22:745–763. 10.1146/annurev.immunol.22.012703.104702 15032595

[bib113] San-Miguel, J., B. Dhakal, K. Yong, A. Spencer, S. Anguille, M.V. Mateos, C. Fernández de Larrea, J. Martínez-López, P. Moreau, C. Touzeau, . 2023. Cilta-cel or standard care in lenalidomide-refractory multiple myeloma. N. Engl. J. Med. 389:335–347. 10.1056/NEJMoa2303379 37272512

[bib114] Sandoval-Villegas, N., W. Nurieva, M. Amberger, and Z. Ivics. 2021. Contemporary transposon tools: A review and guide through mechanisms and applications of sleeping beauty, piggyBac and Tol2 for genome engineering. Int. J. Mol. Sci. 22:5084. 10.3390/ijms22105084 34064900 PMC8151067

[bib115] Schambach, A., M. Morgan, and B. Fehse. 2021. Two cases of T cell lymphoma following piggybac-mediated CAR T cell therapy. Mol. Ther. 29:2631–2633. 10.1016/j.ymthe.2021.08.013 34433080 PMC8417502

[bib116] Scholler, J., T.L. Brady, G. Binder-Scholl, W.T. Hwang, G. Plesa, K.M. Hege, A.N. Vogel, M. Kalos, J.L. Riley, S.G. Deeks, . 2012. Decade-long safety and function of retroviral-modified chimeric antigen receptor T cells. Sci. Transl. Med. 4:132ra53. 10.1126/scitranslmed.3003761 PMC4368443 22553251

[bib165] Schultz, K.L.W. 2021. Summary Basis for Regulatory Action: lisocabtagene maraleucel. https://fda.report/media/146242/Summary+Basis+for+Regulatory+Action+-+BREYANZI.pdf

[bib117] Schultz, L.M., C. Baggott, S. Prabhu, H.L. Pacenta, C.L. Phillips, J. Rossoff, H.E. Stefanski, J.A. Talano, A. Moskop, S.P. Margossian, . 2022. Disease burden affects outcomes in pediatric and young adult B-cell lymphoblastic leukemia after commercial tisagenlecleucel: A pediatric real-world chimeric antigen receptor consortium report. J. Clin. Oncol. 40:945–955. 10.1200/JCO.20.03585 34882493 PMC9384925

[bib118] Schuster, S.J., M.R. Bishop, C.S. Tam, E.K. Waller, P. Borchmann, J.P. McGuirk, U. Jäger, S. Jaglowski, C. Andreadis, J.R. Westin, . 2019. Tisagenlecleucel in adult relapsed or refractory diffuse large B-cell lymphoma. N. Engl. J. Med. 380:45–56. 10.1056/NEJMoa1804980 30501490

[bib119] Schuster, S.J., C.S. Tam, P. Borchmann, N. Worel, J.P. McGuirk, H. Holte, E.K. Waller, S. Jaglowski, M.R. Bishop, L.E. Damon, . 2021. Long-term clinical outcomes of tisagenlecleucel in patients with relapsed or refractory aggressive B-cell lymphomas (JULIET): A multicentre, open-label, single-arm, phase 2 study. Lancet Oncol. 22:1403–1415. 10.1016/S1470-2045(21)00375-2 34516954

[bib120] Seet, C.S., C. He, M.T. Bethune, S. Li, B. Chick, E.H. Gschweng, Y. Zhu, K. Kim, D.B. Kohn, D. Baltimore, . 2017. Generation of mature T cells from human hematopoietic stem and progenitor cells in artificial thymic organoids. Nat. Methods. 14:521–530. 10.1038/nmeth.4237 28369043 PMC5426913

[bib121] Sehgal, A., D. Hoda, P.A. Riedell, N. Ghosh, M. Hamadani, G.C. Hildebrandt, J.E. Godwin, P.M. Reagan, N. Wagner-Johnston, J. Essell, . 2022. Lisocabtagene maraleucel as second-line therapy in adults with relapsed or refractory large B-cell lymphoma who were not intended for haematopoietic stem cell transplantation (PILOT): An open-label, phase 2 study. Lancet Oncol. 23:1066–1077. 10.1016/S1470-2045(22)00339-4 35839786

[bib122] Shah, N.N., B.D. Johnson, D. Schneider, F. Zhu, A. Szabo, C.A. Keever-Taylor, W. Krueger, A.A. Worden, M.J. Kadan, S. Yim, . 2020. Bispecific anti-CD20, anti-CD19 CAR T cells for relapsed B cell malignancies: A phase 1 dose escalation and expansion trial. Nat. Med. 26:1569–1575. 10.1038/s41591-020-1081-3 33020647

[bib123] Shah, N.N., H. Qin, B. Yates, L. Su, H. Shalabi, M. Raffeld, M.A. Ahlman, M. Stetler-Stevenson, C. Yuan, S. Guo, . 2019a. Clonal expansion of CAR T cells harboring lentivector integration in the CBL gene following anti-CD22 CAR T-cell therapy. Blood Adv. 3:2317–2322. 10.1182/bloodadvances.2019000219 31387880 PMC6693002

[bib124] Shah, N.N., F. Zhu, D. Schneider, W. Krueger, A. Worden, W.L. Longo, M. Hamadani, T.S. Fenske, B. Dropulic, R. Orentas, . 2019b. Fresh versus cryopreserved/thawed bispecific anti-CD19/CD20 CAR-T cells for relapsed, refractory non-hodgkin lymphoma. Blood. 134:4465. 10.1182/blood-2019-125328

[bib125] Shahid, S., K. Ramaswamy, J. Flynn, A. Mauguen, K. Perica, J.H. Park, C.J. Forlenza, N.N. Shukla, P.G. Steinherz, S.P. Margossian, . 2022. Impact of bridging chemotherapy on clinical outcomes of CD19-specific CAR T Cell therapy in children/young adults with relapsed/refractory B Cell acute lymphoblastic leukemia. Transplant Cell Ther. 28:72 e71–72 e78. 10.1016/j.jtct.2021.11.014 PMC9361393 34852305

[bib126] Shao, L., R. Shi, Y. Zhao, H. Liu, A. Lu, J. Ma, Y. Cai, T. Fuksenko, A. Pelayo, N.N. Shah, . 2022. Genome-wide profiling of retroviral DNA integration and its effect on clinical pre-infusion CAR T-cell products. J. Transl. Med. 20:514. 10.1186/s12967-022-03729-5 36348415 PMC9644589

[bib127] Smith, T.T., S.B. Stephan, H.F. Moffett, L.E. McKnight, W. Ji, D. Reiman, E. Bonagofski, M.E. Wohlfahrt, S.P.S. Pillai, and M.T. Stephan. 2017. In situ programming of leukaemia-specific T cells using synthetic DNA nanocarriers. Nat. Nanotechnol. 12:813–820. 10.1038/nnano.2017.57 28416815 PMC5646367

[bib128] Sommermeyer, D., M. Hudecek, P.L. Kosasih, T. Gogishvili, D.G. Maloney, C.J. Turtle, and S.R. Riddell. 2016. Chimeric antigen receptor-modified T cells derived from defined CD8+ and CD4+ subsets confer superior antitumor reactivity in vivo. Leukemia. 30:492–500. 10.1038/leu.2015.247 26369987 PMC4746098

[bib129] Sperling, A.S., B.A. Derman, S. Nikiforow, S. Im, S. Ikegawa, R.H. Prabhala, D. Hernandez Rodriguez, Y. Li, D.S. Quinn, D. Pearson, . 2023. Updated phase I study results of PHE885, a T-Charge manufactured BCMA-directed CAR-T cell therapy, for patients (pts) with r/r multiple myeloma (RRMM). J. Clin. Oncol. 16:8004. 10.1200/JCO.2023.41.16_suppl.8004

[bib130] St Martin, Y., J.K. Franz, M.E. Agha, and H.M. Lazarus. 2023. Failure of CAR-T cell therapy in relapsed and refractory large cell lymphoma and multiple myeloma: An urgent unmet need. Blood Rev. 60:101095. 10.1016/j.blre.2023.101095 37173224

[bib131] Stadtmauer, E.A., J.A. Fraietta, M.M. Davis, A.D. Cohen, K.L. Weber, E. Lancaster, P.A. Mangan, I. Kulikovskaya, M. Gupta, F. Chen, . 2020. CRISPR-engineered T cells in patients with refractory cancer. Science. 367:eaba7365. 10.1126/science.aba7365 32029687 PMC11249135

[bib132] Stroncek, D.F., J. Ren, D.W. Lee, M. Tran, S.E. Frodigh, M. Sabatino, H. Khuu, M.S. Merchant, and C.L. Mackall. 2016. Myeloid cells in peripheral blood mononuclear cell concentrates inhibit the expansion of chimeric antigen receptor T cells. Cytotherapy. 18:893–901. 10.1016/j.jcyt.2016.04.003 27210719 PMC4898642

[bib133] Teoh, J., and L.F. Brown. 2022. Developing lisocabtagene maraleucel chimeric antigen receptor T-cell manufacturing for improved process, product quality and consistency across CD19^+^ hematologic indications. Cytotherapy. 24:962–973. 10.1016/j.jcyt.2022.03.013 35610089

[bib134] Themeli, M., C.C. Kloss, G. Ciriello, V.D. Fedorov, F. Perna, M. Gonen, and M. Sadelain. 2013. Generation of tumor-targeted human T lymphocytes from induced pluripotent stem cells for cancer therapy. Nat. Biotechnol. 31:928–933. 10.1038/nbt.2678 23934177 PMC5722218

[bib135] Tong, C., Y. Zhang, Y. Liu, X. Ji, W. Zhang, Y. Guo, X. Han, D. Ti, H. Dai, C. Wang, . 2020. Optimized tandem CD19/CD20 CAR-engineered T cells in refractory/relapsed B-cell lymphoma. Blood. 136:1632–1644. 10.1182/blood.2020005278 32556247 PMC7596761

[bib136] Trainor, N., K.A. Purpura, K. Middleton, K. Fargo, L. Hails, M. Vicentini-Hogan, C. McRobie, R. Daniels, P. Densham, P. Gardin, . 2023. Automated production of gene-modified chimeric antigen receptor T cells using the cocoon platform. Cytotherapy. S1465-3249:01013–01017. 10.1016/j.jcyt.2023.07.012 37690020

[bib137] Tsuchida, C.A., N. Brandes, R. Bueno, M. Trinidad, T. Mazumder, B. Yu, B. Hwang, C. Chang, J. Liu, Y. Sun, . 2023. Mitigation of chromosome loss in clinical CRISPR-Cas9-engineered T cells. Cell. 186:4567–4582.e20. 10.1016/j.cell.2023.08.041 37794590 PMC10664023

[bib138] Tyagarajan, S., T. Spencer, and J. Smith. 2019. Optimizing CAR-T cell manufacturing processes during pivotal clinical trials. Mol. Ther. Methods Clin. Dev. 16:136–144. 10.1016/j.omtm.2019.11.018 31988978 PMC6970133

[bib139] Ueda, T., S. Shiina, S. Iriguchi, S. Terakura, Y. Kawai, R. Kabai, S. Sakamoto, A. Watanabe, K. Ohara, B. Wang, . 2023. Optimization of the proliferation and persistency of CAR T cells derived from human induced pluripotent stem cells. Nat. Biomed. Eng. 7:24–37. 10.1038/s41551-022-00969-0 36509913 PMC9870784

[bib140] van der Stegen, S.J.C., P.L. Lindenbergh, R.M. Petrovic, H. Xie, M.P. Diop, V. Alexeeva, Y. Shi, J. Mansilla-Soto, M. Hamieh, J. Eyquem, . 2022. Generation of T-cell-receptor-negative CD8αβ-positive CAR T cells from T-cell-derived induced pluripotent stem cells. Nat. Biomed. Eng. 6:1284–1297. 10.1038/s41551-022-00915-0 35941192 PMC9669107

[bib141] Vigdal, T.J., C.D. Kaufman, Z. Izsvák, D.F. Voytas, and Z. Ivics. 2002. Common physical properties of DNA affecting target site selection of sleeping beauty and other Tc1/mariner transposable elements. J. Mol. Biol. 323:441–452. 10.1016/S0022-2836(02)00991-9 12381300

[bib142] Wang, M., J. Munoz, A. Goy, F.L. Locke, C.A. Jacobson, B.T. Hill, J.M. Timmerman, H. Holmes, S. Jaglowski, I.W. Flinn, . 2020. KTE-X19 CAR T-cell therapy in relapsed or refractory mantle-cell lymphoma. N. Engl. J. Med. 382:1331–1342. 10.1056/NEJMoa1914347 32242358 PMC7731441

[bib143] Wang, V., M. Gauthier, V. Decot, L. Reppel, and D. Bensoussan. 2023. Systematic review on CAR-T cell clinical trials up to 2022: Academic center input. Cancers. 15:1003. 10.3390/cancers15041003 36831349 PMC9954171

[bib144] Wang, X., O. Borquez-Ojeda, J. Stefanski, F. Du, J. Qu, J. Chaudhari, K. Thummar, M. Zhu, L.B. Shen, M. Hall, . 2021a. Depletion of high-content CD14^+^ cells from apheresis products is critical for successful transduction and expansion of CAR T cells during large-scale cGMP manufacturing. Mol. Ther. Methods Clin. Dev. 22:377–387. 10.1016/j.omtm.2021.06.014 34514029 PMC8411225

[bib145] Wang, Z., N. Li, K. Feng, M. Chen, Y. Zhang, Y. Liu, Q. Yang, J. Nie, N. Tang, X. Zhang, . 2021b. Phase I study of CAR-T cells with PD-1 and TCR disruption in mesothelin-positive solid tumors. Cell. Mol. Immunol. 18:2188–2198. 10.1038/s41423-021-00749-x 34381179 PMC8429583

[bib146] Wang, X., L.L. Popplewell, J.R. Wagner, A. Naranjo, M.S. Blanchard, M.R. Mott, A.P. Norris, C.W. Wong, R.Z. Urak, W.C. Chang, . 2016. Phase 1 studies of central memory-derived CD19 CAR T-cell therapy following autologous HSCT in patients with B-cell NHL. Blood. 127:2980–2990. 10.1182/blood-2015-12-686725 27118452 PMC4911862

[bib147] Wang, X., and I. Rivière. 2022. Manufacturing of CAR-T cells: The assembly line. In Gene and Cellular Immunotherapy for Cancer. A. Ghobadi, andJ.F. DiPersio, editor. Humana, Cham, Switzerland. 121–139. 10.1007/978-3-030-87849-8_8

[bib148] Weber, E.W., K.R. Parker, E. Sotillo, R.C. Lynn, H. Anbunathan, J. Lattin, Z. Good, J.A. Belk, B. Daniel, D. Klysz, . 2021. Transient rest restores functionality in exhausted CAR-T cells through epigenetic remodeling. Science. 372:eaba1786. 10.1126/science.aba1786 33795428 PMC8049103

[bib149] Yang, J., J. He, X. Zhang, J. Li, Z. Wang, Y. Zhang, L. Qiu, Q. Wu, Z. Sun, X. Ye, . 2022. Next-day manufacture of a novel anti-CD19 CAR-T therapy for B-cell acute lymphoblastic leukemia: First-in-human clinical study. Blood Cancer J. 12:104. 10.1038/s41408-022-00694-6 35798714 PMC9262977

[bib161] Ye, Z. 2022. Summary Basis for Regulatory Action: Ciltacabtagene Autoleucel. U.S. Food & Drug Administration. https://www.fda.gov/media/156999/download

[bib150] Yoon, S.H., J.M. Lee, H.I. Cho, E.K. Kim, H.S. Kim, M.Y. Park, and T.G. Kim. 2009. Adoptive immunotherapy using human peripheral blood lymphocytes transferred with RNA encoding Her-2/neu-specific chimeric immune receptor in ovarian cancer xenograft model. Cancer Gene Ther. 16:489–497. 10.1038/cgt.2008.98 19096447

[bib151] Zah, E., E. Nam, V. Bhuvan, U. Tran, B.Y. Ji, S.B. Gosliner, X. Wang, C.E. Brown, and Y.Y. Chen. 2020. Systematically optimized BCMA/CS1 bispecific CAR-T cells robustly control heterogeneous multiple myeloma. Nat. Commun. 11:2283. 10.1038/s41467-020-16160-5 32385241 PMC7210316

[bib152] Zhang, D.K.Y., K. Adu-Berchie, S. Iyer, Y. Liu, N. Cieri, J.M. Brockman, D. Neuberg, C.J. Wu, and D.J. Mooney. 2023. Enhancing CAR-T cell functionality in a patient-specific manner. Nat. Commun. 14:506. 10.1038/s41467-023-36126-7 36720856 PMC9889707

[bib153] Zhang, Y., Z. Zhang, Y. Ding, Y. Fang, P. Wang, W. Chu, Z. Jin, X. Yang, J. Wang, J. Lou, and Q. Qian. 2021. Phase I clinical trial of EGFR-specific CAR-T cells generated by the piggyBac transposon system in advanced relapsed/refractory non-small cell lung cancer patients. J. Cancer Res. Clin. Oncol. 147:3725–3734. 10.1007/s00432-021-03613-7 34032893 PMC11801842

[bib154] Zhao, Y., E. Moon, C. Carpenito, C.M. Paulos, X. Liu, A.L. Brennan, A. Chew, R.G. Carroll, J. Scholler, B.L. Levine, . 2010. Multiple injections of electroporated autologous T cells expressing a chimeric antigen receptor mediate regression of human disseminated tumor. Cancer Res. 70:9053–9061. 10.1158/0008-5472.CAN-10-2880 20926399 PMC2982929

[bib155] Zhao, Y., Z. Zheng, C.J. Cohen, L. Gattinoni, D.C. Palmer, N.P. Restifo, S.A. Rosenberg, and R.A. Morgan. 2006. High-efficiency transfection of primary human and mouse T lymphocytes using RNA electroporation. Mol. Ther. 13:151–159. 10.1016/j.ymthe.2005.07.688 16140584 PMC1473967

[bib156] Zhu, F., N. Shah, H. Xu, D. Schneider, R. Orentas, B. Dropulic, P. Hari, and C.A. Keever-Taylor. 2018. Closed-system manufacturing of CD19 and dual-targeted CD20/19 chimeric antigen receptor T cells using the CliniMACS Prodigy device at an academic medical center. Cytotherapy. 20:394–406. 10.1016/j.jcyt.2017.09.005 29287970

